# Multitargeting nature of muscarinic orthosteric agonists and antagonists

**DOI:** 10.3389/fphys.2022.974160

**Published:** 2022-09-06

**Authors:** Jaromir Myslivecek

**Affiliations:** Institute of Physiology, 1^st^ Faculty of Medicine, Charles University, Prague, Czechia

**Keywords:** muscarinic receptors, multitarget, muscarinic agonist, muscarinic antagonist, allosteric, orthosteric

## Abstract

Muscarinic receptors (mAChRs) are typical members of the G protein-coupled receptor (GPCR) family and exist in five subtypes from M_1_ to M_5_. Muscarinic receptor subtypes do not sufficiently differ in affinity to orthosteric antagonists or agonists; therefore, the analysis of receptor subtypes is complicated, and misinterpretations can occur. Usually, when researchers mainly specialized in CNS and peripheral functions aim to study mAChR involvement in behavior, learning, spinal locomotor networks, biological rhythms, cardiovascular physiology, bronchoconstriction, gastrointestinal tract functions, schizophrenia, and Parkinson’s disease, they use orthosteric ligands and they do not use allosteric ligands. Moreover, they usually rely on manufacturers’ claims that could be misleading. This review aimed to call the attention of researchers not deeply focused on mAChR pharmacology to this fact. Importantly, limited selective binding is not only a property of mAChRs but is a general attribute of most neurotransmitter receptors. In this review, we want to give an overview of the most common off-targets for established mAChR ligands. In this context, an important point is a mention the tremendous knowledge gap on off-targets for novel compounds compared to very well-established ligands. Therefore, we will summarize reported affinities and give an outline of strategies to investigate the subtype’s function, thereby avoiding ambiguous results. Despite that, the multitargeting nature of drugs acting also on mAChR could be an advantage when treating such diseases as schizophrenia. Antipsychotics are a perfect example of a multitargeting advantage in treatment. A promising strategy is the use of allosteric ligands, although some of these ligands have also been shown to exhibit limited selectivity. Another new direction in the development of muscarinic selective ligands is functionally selective and biased agonists. The possible selective ligands, usually allosteric, will also be listed. To overcome the limited selectivity of orthosteric ligands, the recommended process is to carefully examine the presence of respective subtypes in specific tissues via knockout studies, carefully apply “specific” agonists/antagonists at appropriate concentrations and then calculate the probability of a specific subtype involvement in specific functions. This could help interested researchers aiming to study the central nervous system functions mediated by the muscarinic receptor.

## 1 Introduction

It is widely accepted that many neurotransmitters bind to different receptor types. As an example, glutamate binds to metabotropic glutamate receptors (mGlu) and to a group of ligand-gated ion channels (N-methyl-D-aspartate (NMDA), α-amino-3-hydroxy-5-methyl-4-isoxazole propionic acid (AMPA) and kainite receptors). Serotonin binds mainly to G protein-coupled receptors (GPCRs), but 5-HT_3_ receptors are ligand-gated ion channels. Similarly, acetylcholine (ACh) binds to nicotinic receptors (NRs), which are ligand-gated ion channels, and to muscarinic receptors (mAChRs), which are typical G protein-coupled receptors.

Commonly, less is known about the properties of ligands used in research on the central nervous system (CNS) and peripheral tissue functions. In this review, we will focus on mAChRs. As an example, the recent translational research of human epithelial ovarian carcinoma have described the role of M_2_ mAChRs in M_2_ cell growth and survival (Taggi et al., 2022). The authors have used arecaidine propargyl ester as M_2_ mAChR preferential agonist. However, as it can be deduced from Table 1, arecaidine propargyl ester binds with similar affinity to M_2_-M_4_ mAChRs, slightly better that to M_1_ mAChRs. These conclusions could be therefore misleading.

**TABLE 1:** Effects of orthosteric agonists (+endogenous ligand) on muscarinic receptors and other targets. The compounds are listed alphabetically. The numbers indicate pKi values. Please note that a higher pKi indicates higher affinity. Unavailable data are shown as blank spaces. The affinities of nicotinic receptors are not divided among specific subunits. The affinities of other, noncholinergic targets are divided into nanomolar (pKi>6) and micromolar (pKi<6) groups. The categorization of pKi is compromise between extremely large Table and fast orientation how much are other targets are affected in specific concentration of ligand used (see also examples in Chapter 5). Data are from or adapted from (Pascuzzo et al., 1984; Eglen and Whiting, 1987; Buckley et al., 1989; Buckley et al., 1990; Hudkins et al., 1991; Wess et al., 1991; Bolden et al., 1992; Kashihara et al., 1992; Sowell et al., 1992; Doods et al., 1993; Shen et al., 1993; Stanton et al., 1993; Doods et al., 1994; Ferrari-Dileo et al., 1994; Kleinschroth et al., 1995; Waelbroeck et al., 1996; McKenna et al., 1997; Boess et al., 1998; Bolognesi et al., 1998; Cantí et al., 1998; Caulfield and Birdsall, 1998; Lazareno et al., 1998; Choppin et al., 1999; Sánchez and Hyttel, 1999; Eglen and Nahorski, 2000; Kozlowski et al., 2000; Lazareno et al., 2000; Becerra et al., 2001; Dhein et al., 2001; Huang et al., 2001; Lockhart et al., 2001; Apelt et al., 2002; Carlsson et al., 2002; Harvey et al., 2002; Cheng et al., 2002; Lazareno et al., 2002; Böhme et al., 2003; Samochocki et al., 2003; Sur et al., 2003; Jakubik et al., 2004; Kobayashi et al., 2004; Wang et al., 2004; Ghoneim et al., 2006; Spalding et al., 2006; Butini et al., 2008; Langmead et al., 2008; Bridges et al., 2009; Heinrich et al., 2009; Prat et al., 2009; von Coburg et al., 2009; Bridges et al., 2010a; Harada et al., 2010; Hern et al., 2010; Lange et al., 2010; Rook et al., 2010; Sinha et al., 2010; Watt et al., 2011; Daval et al., 2012; Samadi et al., 2012; Sykes et al., 2012; Arunotayanun et al., 2013; Nenasheva et al., 2013; Salmon et al., 2013; Croy et al., 2016; Gaulton et al., 2016; Alexander S. P. et al., 2017; Alexander S. P. H. et al., 2017; Chen et al., 2017; Carr et al., 2018; Hegde et al., 2018; Broad et al., 2019; Myslivecek, 2019; Xu et al., 2019; Olianas et al., 2020; Okimoto et al., 2021) and the IUPHAR/BPS Guide to Pharmacology (www.guidetophamacology.org). For specific ligand references see text. The activity represents the main effect of a specific ligand.

Target	Muscarinic receptors					Nicotinic receptors	Cholinester-ases (ChEs) AChE/BChE	Other targets	
	M_1_	M_2_	M_3_	M_4_	M_5_			Nanomolar (pKi > 6)	Micromolar (pKi < 6)
Drug	Endogenous ligand								
Acetylcholine	4.3–4.9	6.4	5.6	4.5–5.6	6.1	4.06–8.77			
Orthosteric agonists									
77-LH-28-1	8.7	5.5 ± 0.1		5.9 ± 0.2	5.8 ± 0.4			D_2_DR, 5-HT_2B_	5HT_2C_
AC-260584 (*)	7.39 (5.9)	5.0–6.16	5.23	5.0	6.0			D_2_DR	5HT_2C_, 5-HT_2B_
AC-42 (*)	6.2 (6.2)	5.76–6.01	5.55–6.0	5.85–6.0	5.35–6.0	6.0	6.0	α_1A,B,D_-AR, α_2A,B_-AR, D_2_DR, D_4_DR, H_1_R, 5-HT_1A_,	A_1_ R, A_2A_ R, A_3_ R, β_1,2_-AR, CB_1_ R, CCK R, GR, ChT, MAO, NPY R, SERT,
(-)-aceclidine^#^	5.4[5.6–5.7]	6.2–6.4[5.1]	5.7[5.1]	5.4[5.0]	5.5[4.8]				
arecaidine propargyl ester	6.4	5.7	5.7	5.9					
Arecoline	5.7	5.2	5.4	5.5		6.57–6.65			CACNA1C
Bethanechol	4.0	4.0	4.2	4.0					
butylthio-TZTP	(PET)							σ_1_R (PET)	
Carbachol	3.2–5.3	4.2–5.7	4.0–4.4	4.3–4.9	4.9	4.18–6.12	not cleaved		AANAT
cevimeline	5.3	6.1	5.6	6.0					
furtrethonium	4.1	4.5	4.1	4.3					
Iperoxo	5.67–10.1	9.8		9.52 ± 0.81					
LY-593039	6.21–7.63	6.05–7.54	>5.0	>5.0	>5.0			D_2_DR, 5-HT_2B_	5HT_2C_
methacholine	6.4	7.2	6.9	5.8					AANAT
Methylfurmethide	4.6	4.9	4.6	4.7		2.0			
milameline^$^	5.5		5.4	5.1	4.8				
NNC 11-1314	7.4	7.2	7.1–7.7	7.3	7.8				
NNC 11-1585	9.9	10.1	8.3	8.6	8.3				
NNC 11-1607	8.6	8.2	8.1	8.1	8.2				
oxotremorine	5.5–6.0	5.0–6.6	5.3	5.2	5.1–7.26		5.82–8.77		AANAT
oxotremorine-M	5.1–5.6	4.9	5.1	5.2					
pentylthio-TZTP	8.6	7.9	8.1	8.7					
pilocarpine	4.9–5.1	4.9	5.1	5.2	5.0				
sabcomeline (SB-202026)	6.7		7.0	7.2	7.1				
SPP1	7.67	6.89	5.10	6.94	6.71				
xanomeline (*)	6.7–7.9 (7.8 ± 0.1)	6.9–7.4	7.2–7.4	7.4–7.7	6.7–7.4			5HT_1A,1B,1D,1F_	5-HT_6_, 5-ht_1e_
								5-HT_2A,2B,2C,_ 5-HT_7_	
(-)YM796	4.3–4.8								
(±)YM796	4.1–4.7								

5-HT, serotonin receptors (with specific subtypes); AANAT, serotonin N-acetyltransferase (arylalkylamine N-acetyltransferase); A_1_R, adenosine A_1_ receptor; A_2A_R, adenosine A_2A_ receptor; A_3_R, adenosine A_3_ receptor; AR, androgen receptors; ASL, argininosuccinate lyase; α_1_-AR, α_1_-adrenoceptors (with specific subtypes); α_2_-AR, α_2_-adrenoceptors (with specific subtypes); β-AR, β-adrenoceptors (with specific subtypes); BTLP, brain tumor-like proteins; CANCA1C, voltage-dependent L-type calcium channel subunit alpha-1C subunit; Ca_v_1.2, voltage-gated L-type calcium channel alpha-1C subunit; Ca^2+^/calmodulin PK II, calcium/calmodulin protein kinase II; CB R, cannabinoid receptors; CCK R, cholecystokinin receptor; cGMP-PK, cGMP dependent protein kinase; ChT, high-affinity choline transporter; CYP2C19, CYP2C9, cytochrome P450 enzymes; δ-OR, δ-opioid receptor; D_1,2,3,4,5_DR, dopamine D_1,2,3,4,5_ receptors; DAT, dopamine transporter; DAPK1, death-associated protein kinase 1, ET_A_R, endothelin ET_A_ receptors; GluN1/GluN2A subunits of NMDA glutamate receptor; GlyR, glycine receptor (subunits in parentheses); GR, glucocorticoid receptor; H_1_R, H_2_R, H_3_R, histamine receptors 1, 2, and 3; K_ir_, potassium inward rectifier; K_v_1.7, voltage-gated potassium channel 1.7; K_v_11.1, rapid delayed inward rectifying potassium current; K_v_7.1, voltage-gated potassium channel; K_v_4.3, voltage-gated potassium channel subunit K_v_4.3; LPA, Lysophospholipid receptors; MAO_A,B_, monoaminoxidase A, B; MAPK, mitogen-activated protein kinase; MC_4_R, melanocortin receptor 4; MLCK, myosin light chain kinase; MT_1A_R, melatonin receptor 1A; NMDA GluN1/GluN2A, subunits of NMDA receptors; NA, noradrenaline; Na_V_, sodium channels (batrachotoxin site); Na_v_1.5, sodium channel protein type V; OCT-2, organic cation transporter 2; TRPV, transient receptor potential vanilloid ion channel; SLC22A1, solute carrier 22, type 1; NET, norepinephrine transporter, neurotrophic rectyrK1/NGF receptor Trk-A, neurotrophic receptor tyrosine kinase 1/Nerve growth factor receptor Trk-A; NPY R, neuropeptide Y receptor; σR, sigma nonopioid receptor; SERT, serotonin (5-HT) transporter; ser/thr kinase 3, serin/threonine kinase 3; TAS2R46, TAS2R10, Taste receptor type 2 (member 46, 10), TRα; TRβ1, thyroid receptors subtypes; VAChT, vesicular acetylcholine transporter.

*pIC_50_ given instead of pK_i_. ^#^pEC_50_ given instead of pK_i_. (*): also acts as an allosteric modulator. The values in parentheses are the values for allosteric binding. The values in brackets are the values when ligand also act as partial agonist. (PET) the selectivity was determined using PET study.

It could be surprising to mention that majority of GPCRs can be activated by multiple ligands (Vass et al., 2018), e.g., 1312 potentially active ligands (searched from ChEMBL database (Gaulton et al., 2016), with K_i_<1 µmol/L) exist for M_1_ mAChRs, of which 930 are active at other GPCRs (i.e., 70%). Similarly, among 1336 potentially active ligands for M_2,_ 974 are active at other GPCRs (i.e., 73%), and the corresponding numbers for M_3_ mAChRs and M_4_ mAChRs are 1384 vs. 855 (i.e., 62%) and 466 vs. 387 (i.e., 83%), respectively. Thus, these authors talk about the interactome.

In general, the off-target is defined as targets (proteins or other molecules in the body) other than those for which the drug was meant to bind. This can lead to unexpected side effects that may be both harmful and positive. Learning about the off-target effects of drugs may help in drug development. Alternatively, these drugs are designated as multitargeting drugs.

Muscarinic receptors can provide good examples of ligands exhibiting multitarget properties. Typically, mAChR subtypes do not greatly differ in their affinity to orthosteric antagonists (Farar and Myslivecek, 2016; Valuskova et al., 2018a; Myslivecek, 2019) or agonists (Alexander S. P. et al., 2017), which complicates the analysis determining receptor subtypes involved in the specific function and misinterpretations can occur. Thus, the exact role of a specific muscarinic subtype is an important issue that is usually not precisely considered when studying its functions.

There are five mAChR subtypes (M_1_-M_5_). They can be classified into the even-numbered subtypes (M_2_,M_4_) that preferentially couple to heterotrimeric G_i_ proteins and the odd-numbered (M_1_,_3_,_5_) that preferentially couple to heterotrimeric G_q_ proteins. In detail, odd-numbered mAChRs are more similar in amino acid sequence composition (see Section 6) between each other than this group and the other group (even-numbered mAChRs).

What is important, mAChRs are involved in many functions, such as learning (Fernández De Sevilla et al., 2021), spinal locomotor networks (Mille et al., 2021), locomotion (Ztaou and Amalric, 2019), biological rhythms (Myslivecek et al., 2017), cardiovascular physiology (Saternos et al., 2017), bronchoconstriction (Kistemaker and Gosens, 2015), and gastrointestinal tract functions (Tobin et al., 2009), and they are also involved in pathologies, such as schizophrenia (Dean and Scarr, 2020) and Parkinson’s disease (Ztaou and Amalric, 2019). Therefore, the choice of appropriate ligand(s) is crucial for mAChR subtype determination in the abovementioned functions.

There are several ways how to discriminate between mAChR subtypes. The first is the choice of appropriate ligand(s) with careful competition binding. This is usually a time and source-consuming issue and sometimes it is not possible (e.g., when it is necessary to see the changes in function depending on mAChR subtype). The second one is the use of the procedure as proposed here. The third possibility is the use of a specific ligand (mostly allosteric) whose number and subtype selectivity are limited to date.

The allosteric ligands have been shown to exhibit selectivity for specific mAChR subtypes (Kruse et al., 2013; Kruse et al., 2014; Bock et al., 2018; Coughlin et al., 2019), providing promising therapeutic effects in diseases connected with mAChRs. However, the search for therapeutically useful ligands should also address additional criteria (i.e., security, bioavailability) beyond subtype specificity. As discussed in Section 4, in most cases, orthosteric ligands bind with higher affinity, and allosteric ligands can provide greater receptor selectivity but are not bound with sufficient affinity. On the other hand, not all orthosteric bind with a higher affinity, and not all allosteric ligands may provide greater receptor selectivity. Bitopic (dualsteric) ligands combine allosteric site selectivity with orthosteric high binding affinity.

Another new direction in the development of selective muscarinic ligands involves functionally selective and biased agonists [for detail, see review (Randáková and Jakubík, 2021)]. However, both allosteric ligands and biased agonists are usually not widely known and thus are not used in mAChR functional studies. On the other hand, allosteric ligands can also target multiple receptors; thus, analyses of mAChR functions using these ligands can be complicated. Additionally, very well-established ligands (with a long history of use in MR research, e.g., atropine) are expected to exhibit more additional targets than less established ligands (with a short history of use or only recently discovered), simply because they are better investigated. Another aspect that complicates the determination of appropriate receptor subtypes in specific tissues is the presence of multiple mAChR subtypes in both periphery tissues, e.g., the retina (Ruan et al., 2021) and heart (Myslivecek et al., 2008a; Myslivecek et al., 2008b), and CNS tissues, e.g., the striatum (Felder et al., 2018), hippocampus (Ohno-Shosaku et al., 2003) and other tissues. In the CNS, the presence of multiple mAChR subtypes on the same structure (presynaptic or postsynaptic) thus complicates the identification of target structures. In the brain, some regions have targets being co-expressed, whereas regions have only one target or subtype predominantly expressed. This can be also relevant if a ligand is targeting regions in the brain and only one region is of interest. The number of receptors (B_max_) also plays a role in receptor interactions.

One can argue that it is possible to use antibodies against specific mAChR subtypes; however, antibodies are very often not specific to the declared targets and can reveal the presence of specific subtypes, even when using mAChR subtype-specific knockout (KO) (Pradidarcheep and Michel, 2016).

Here, we focus on the multiple-target binding of widely used muscarinic drugs that are usually considered subtype-specific. We mainly focus on orthosteric ligands because of their broad usage. We also will emphasize the allosteric drugs that have been shown to be subtype-selective.

Usually, but not in all cases, the orthosteric agonists need micromolar concentration to activate the receptors (see Table 1). The concentration of agonists in signaling assays will depend on receptor expression, receptor reserve, and the amplification of the signaling assay. The antagonists can block the receptors with nanomolar concentration. The common problem in the activation/blockade of mAChR is that the ligand is usually used in high concentrations (see examples in Section 5). Thus, we tried to search available databases (PubMed, Web of Science) for all possible molecules that are able to bind to other targets (proteins or other molecules in the body) than mAChRs. The drugs were divided according to the affinity of off-targets in two groups (nanomolar and micromolar). The reader can then compare the concentration used with the target and judge if this off-target is affected or not.

We show here that most mAChR orthosteric ligands that are usually considered subtype-specific can bind to other receptors, which can be important when interpreting the roles of mAChR subtypes in specific events in both periphery and CNS tissues. This review aimed to call the attention of researchers not deeply focused on mAChR pharmacology to this fact. A very simple search of PubMed (accessed on 20 June 2022) “pirenzepine and M_1_ selective” reached 984 references—i.e., publications in which pirenzepine could be potentially considered as M_1_ selective, which is far from true (see below). In fact, the number of these publications could be lower as not always pirenzepine is considered as M_1_ selective (just coincidence can occur). However, in most of these references is pirenzepine considered to be M_1_ selective. Similarly, a search on “AF-DX 116 and M_2_ selective” (accessed on 20 June 2022) reached 313 results—thus, these publications potentially consider AF-DX 116 as M_2_ selective. The data reviewed here, however, do not report this selectivity.

Additionally, it is necessary to stress that multitarget drug interactions can be considered an advantage in drug effects, as they allow drugs to target more receptors and thus activate more signaling pathways (typically involving sigma receptors) (Maurice and Su, 2009; Abatematteo et al., 2021), which can decrease the required dose of a given drug and produce additive effects with the use of one drug.

## 2 Muscarinic receptor orthosteric agonists

### 2.1 Established agonists of mAChRs and their off-targets

The endogenous ligand of mAChRs, ACh, also binds to NRs. Other mAChR agonists are able to activate multiple targets, at least including all mAChR subtypes. This can be a problem when one tries to discriminate between effects on specific receptor subtypes. The widely used mAChR agonist carbachol (carbamylcholine, see Table 1) also binds to NRs (Bolchi et al., 2013) and is not hydrolyzable by cholinesterases, which can be both advantageous and disadvantageous when studying associated effects. The presence of NRs on presynaptic or postsynaptic membranes can affect neurotransmission.

In general, mAChR agonists (and partial agonists, see Table 1) are not subtype-selective. Concretely, pilocarpine, bethanechol, oxotremorine, arecoline, oxotremorine-M, xanomeline, cevimeline, methacholine, iperoxo, methylfurmetide, pentylthio-TZTP, sabcomeline, arecaidine propargyl ester, milameline, furtrethonium (furmethid), aceclidine and relatively newly synthesized compounds, such as SPP1, NNC 11-1585, NNC 11-1607, and NNC 11-1314, are nonselective with respect to a single mAChR subtype (Alexander S. P. et al., 2017; Broad et al., 2019), although they can express higher selectivity for some subtypes, as is the case for NNC 11-1585, which is more selective for M_1_ and M_2_ mAChRs than for other mAChR subtypes. Some of them, although declared as subtype-selective, do not reveal specific subtype selectivity. For example, oxotremorine, which is usually considered M_1_-selective, activates all muscarinic subtypes with similar affinity (Jakubík et al., 1997). Similarly, pilocarpine (partial agonist), which is often declared M_3_-selective, has comparable effects on all muscarinic receptor subtypes (Alexander S. P. et al., 2017). Also, a relatively newly (2011) synthesized drug, LY-593093, is not M_1_ mAChR selective, as declared but there exists a similar affinity of M_1_ and M_2_ mAChRs [pK_i_ = 6.21, 6.05, for M_1_ and M_2_ mAChRs, respectively, see (Watt et al., 2011)]. In general, it is plausible to expect that orthosteric mAChR agonists will bind to all mAChR subtypes without specific selectivity to one of the five mAChR subtypes (see Table 1 for mAChR subtype pK_i_s: specific agonists are bound with similar affinity to all subtypes). Some drugs, like (-)YM796, (±)YM796 (Wei et al., 1992), AZD6088, LSN3172176 (Nabulsi et al., 2019), butylthio-TZTP (Farde et al., 1996) are not sufficiently documented with respect to selectivity.

In the following paragraphs, the orthosteric muscarinic agonists will be reviewed with respect to other targets. In the 1970s, methylfurmethide was indicated to also produce some effects on NRs (Ochillo et al., 1977). Another example of such a drug is arecoline, which acts on NRs (Ward et al., 1994; Pei et al., 1998) as well as the Ca_v1.2_ calcium current (So et al., 2015), producing inhibitive effects. mAChR agonists additionally target enzyme activities; thus, methacholine, carbachol, and oxotremorine inhibited the activity of serotonin N-acetyltransferase, which is a melatonin-synthesizing enzyme, in bovine pineal explants (Pujito et al., 1991). Oxotremorine has also been shown to inhibit acetylcholinesterase (AChE) activity (Sowell et al., 1992). Some ligands reveal multiple-target actions. This is the case for xanomeline, which is considered a partial agonist for mAChRs (Alexander S. P. et al., 2017). However, this drug also has properties indicating allosteric binding (Jakubik et al., 2004) and binding to many serotonin receptor (SERT) subtypes (5-HT_1A_, 5-HT_1B_, 5-HT_1D_, 5-HT_1F_, 5-HT_2A_-5HT_2C_, 5-HT_6_, and 5-HT_7_) (Watson et al., 1998).

Relatively recently, some new drugs have been synthesized to favor M_1_ mAChR effects over effects on other receptor subtypes (mainly M_3_ mAChRs), including AC-42, AC-260584, 77-LH-28-1, and LY-593039 (Heinrich et al., 2009). However, these drugs can also bind to other neurotransmitter receptors. For example, AC-260584 and 77-LH-28-1 bind similarly to M_1_ mAChRs and D_2_ dopamine receptors and affect 5-HT_2B_ and 5HT_2C_ receptors (Heinrich et al., 2009). LY-593039 also binds to D_2_ dopamine receptors, although slightly less than to M_1_ mAChRs (Heinrich et al., 2009). AC-42 exerts complicated actions on mAChRs and is a typical representative of multitargeting drugs. It acts on M_1_ mAChR as a full agonist and simultaneously functions as a negative allosteric modulator (NAM, see below for an explanation of this term, (Langmead et al., 2006)). On other mAChR subtypes, it behaves as an atypical agonist (Richards and van Giersbergen, 1995). As assessed by broad pharmacology profiling (the compound was tested against 69 different pharmacological targets - receptors, ion channels, transporters, and enzymes), AC-42 was bound with revealed relatively high affinity to histamine H_1_ receptors, D_2_-, and D_4_ dopamine receptors, 5-HT_1A_ serotonin receptors, and α_1A,B,D_- and α_2A,B_-adrenoceptors (Sams et al., 2010). Other targets had a lower affinity to AC-42 than 1 μmol/L.

### 2.2 Established agonists of GPCRs with mAChRs as off-target

Another aspect of mAChR pharmacology is the existence of the interactome (Vass et al., 2018). Although the identification of other neurotransmitters that act on mAChRs is difficult, some data indicate that adenosine can act as an antagonist of some specific mAChR-mediated events. It has been shown that adenosine acts selectively in opposing mechanisms of depolarization of the rat superior cervical ganglion M-current (Connolly and Stone, 1995). Another report described a profound (isobutylthio)adenosine inhibitive effect on mAChR binding (Pankaskie et al., 1985). Recently, nerve growth factor has been shown to physically interact with M_4_ mAChRs (Chen et al., 2021), promoting neuroendocrine differentiation in prostate cancer and producing castration resistance. Droxidopa, which is a synthetic precursor of noradrenaline, used in hypotension treatment, acts also as an antagonist with pK_i_ = 7.1 at M_1_ mAChRs (Croy et al., 2016).

Important findings were obtained with β_3_-adrenoceptor agonist vibegron. It has been shown (Yamada et al., 2021) to have an inhibitory action on ^3^H-NMS (N-methylscopolamine, muscarinic antagonist) binding in different tissue with maximal effect in the heart (pIC_50_ = 6.1 ± 0.16). However, one should consider two facts: first, IC_50_ is not an optimal indicator of the pharmacodynamic properties of a drug (see Section 6), and second, β_3_-adrenoceptor are present in the heart (Benes et al., 2012) which can slightly doubt these results.

## 3 Muscarinic receptor orthosteric antagonists

### 3.1 Established antagonists of mAChRs and their off-targets

Orthosteric antagonists of mAChRs reveal a much wider spectrum of action targets than agonists. Thus, one should be more cautious in interpreting results obtained with the use of mAChR orthosteric antagonists. Typically, although very often declared by manufacturers as “selective ligands,” the vast majority of antagonists bind with similar affinity to more than one mAChR subtype (see Table 2). We have reviewed these properties recently (Myslivecek, 2019). It can be concluded that only a limited number of ligands (e.g., mamba toxins) are selective for one subtype (see Table 4). However, the usage of toxins is not simple, especially *in vivo*, it is difficult to make and expensive to use. These toxins also bind allosterically, rather than only orthosterically (Karlsson et al., 2000). Importantly, the selectivity to M_3_ mAChR was not, according to our knowledge yet proven. The M_3_ mAChR positive allosteric modulator ASP8302 (Okimoto et al., 2021) revealed a similar increase in activity in M_3_ as well as in M_5_ mAChRs (see Table 2).

**TABLE 2:** Effects of orthosteric antagonists on muscarinic receptors and other targets. The compounds are listed alphabetically. The numbers indicate pKi values. Please note that a higher pKi indicates higher affinity. Unavailable data are shown as blank spaces. The affinities of nicotinic receptors are not divided among specific subunits. The affinities of other, noncholinergic targets are divided into nanomolar (pKi>6) and micromolar (pKi<6) groups. The categorization of pKi is compromise between extremely large Table and fast orientation how much are other targets are affected in specific concentration of ligand used (see also examples in Chapter 5). Data are from or adapted from (Pascuzzo et al., 1984; Eglen and Whiting, 1987; Buckley et al., 1989; Buckley et al., 1990; Hudkins et al., 1991; Wess et al., 1991; Bolden et al., 1992; Kashihara et al., 1992; Sowell et al., 1992; Doods et al., 1993; Shen et al., 1993; Stanton et al., 1993; Doods et al., 1994; Ferrari-Dileo et al., 1994; Kleinschroth et al., 1995; Waelbroeck et al., 1996; McKenna et al., 1997; Boess et al., 1998; Bolognesi et al., 1998; Cantí et al., 1998; Caulfield and Birdsall, 1998; Lazareno et al., 1998; Choppin et al., 1999; Sánchez and Hyttel, 1999; Eglen and Nahorski, 2000; Kozlowski et al., 2000; Lazareno et al., 2000; Becerra et al., 2001; Dhein et al., 2001; Huang et al., 2001; Lockhart et al., 2001; Apelt et al., 2002; Carlsson et al., 2002; Harvey et al., 2002; Cheng et al., 2002; Lazareno et al., 2002; Böhme et al., 2003; Samochocki et al., 2003; Sur et al., 2003; Jakubik et al., 2004; Kobayashi et al., 2004; Wang et al., 2004; Ghoneim et al., 2006; Spalding et al., 2006; Butini et al., 2008; Langmead et al., 2008; Bridges et al., 2009; Heinrich et al., 2009; Prat et al., 2009; von Coburg et al., 2009; Bridges et al., 2010a; Harada et al., 2010; Hern et al., 2010; Lange et al., 2010; Rook et al., 2010; Sinha et al., 2010; Watt et al., 2011; Daval et al., 2012; Samadi et al., 2012; Sykes et al., 2012; Arunotayanun et al., 2013; Nenasheva et al., 2013; Salmon et al., 2013; Croy et al., 2016; Gaulton et al., 2016; Alexander S. P. et al., 2017; Alexander S. P. H. et al., 2017; Chen et al., 2017; Carr et al., 2018; Hegde et al., 2018; Broad et al., 2019; Myslivecek, 2019; Xu et al., 2019; Olianas et al., 2020; Okimoto et al., 2021) and the IUPHAR/BPS Guide to Pharmacology (www.guidetophamacology.org). For specific ligand references see text. The activity represents the main effect of a specific ligand.

Target	Muscarinic receptors					Nicotinic receptors	Cholinester-ases (ChEs) AChE/BChE	Other targets	
	M_1_	M_2_	M_3_	M_4_	M_5_			Nanomolar (pKi > 6)	Micromolar (pKi < 6)
3-quinuclidinyl-benzilate	10.29	10.35		10.4					
4-DAMP	8.6–9.2	7.8–8.4	8.9–9.3	8.4–9.4	8.9–9.0				
Aclidinium	10.1–10.2	10.1	10.1–10.2	10.0	9.9				
AE9C90CB		8.6	9.9	9.5	9.5				
AF-DX 116	5.8–6.9	7.1–7.3	5.5–6.6	6.2–7.0	5.4–6.6				
AF-DX 384	7.3–7.5	8.2–9.0	7.2–7.8	8.0–8.7	6.3				
Amitriptyline	7.8	7.9	7.9	8.1	7.8			H_1_R, 5-HT_2A_, 5-HT_2C_, 5-HT_6_, 5-HT_7_, α_1A,B,D_-AR, α_2A,B,C_-AR, D_1_,_2_,_3,5_DR, LPA1, SERT, NET	K_ir_3.2, K_ir_3.4, DAT
AQ-RA 741	7.6–7.8	8.21–8.9	7.4–7.5	7.9–8.2	5.8–6.1				
Atropine	8.5–9.6	9.0–9.1	8.9–9.8	8.7–9.9	9.3–9.7	4.49	9.15–9.46	5-HT_2C_	α_1D,2A_-AR,5-HT_1A_, SLC22A1, glycine receptors
Benzatropine	9.0	8.6	8.89–9.57	8.62–9.48	8.84–8.69			σR, 5-HT_2A,B,C_, H_1_R, DAT, α_1A,B,D_-AR α_2A,B,C_-AR, D_3_DR	SERT, NET, 5-HT_6,_ H_2_R
Biperiden	9.3	8.2	8.4	8.6	8.2				
Clidinium		9.6	9.6						
Darifenacin	7.5–7.8	7.0–7.4	8.4–8.9	7.7–8.0	8.0–8.1			β_1,2_-AR, K_v_11.1	D_2_DR, Na_v_1.5
DAU 5884	9.4 ± 0.04	7.4 ± 0.05	8.8 ± 0.03	8.5 ± 0.02					
dicyclomine	8.61	6.6	9.0	8.3	8.77			5-HT_2A_, 5-HT_2B_, 5-HT_2C_, D_3_DR, σ_1_R, σ_2_R,	Ca_v_1.2, H_2_R, 5-HT_6,_ Kv11.1, Na_V_
(S)-dimetindene	6.7	7.5	6.9	6.5	6.1			H_1_R	
Dosulepin	7.7	7.0	7.4	7.2	7.0			H_1_R, SERT, NET	
Droxidopa	7.1							***NA precursor***	
ethopropazine	8.5	8.1					6.52/6.59		
glycopyrrolate	9.6–10.1*	8.7–9.5*	9.6–9.8*	9.1–10.0	8.9–9.9				
guanylpirenzepine	7.3–7.6	5.3	6.2	6.2	6.8				
hexahydrodifenidol	8.0	6.7	7.8	7.1	7.1				
hexahydrosiladifenidol (HHSiD)	7.4–7.9	6.6–6.8	7.7.-8.0	6.5–7.7	6.8–7.2				
hexocyclium	8.6	7.6	8.9	8.3	8.4				
Himbacine	7.0–7.2	8.0–8.3	6.9–7.4	8.0–8.8	6.1–6.3		4.64		
imipramine		6.9			6.52^#^			α_1A_-AR, D_2_ DR, H_1_ R, 5-HT_2A,C_, NET, SERT	Kv11.1, Kv3.2, Kv3.4, Kv10.1, GluN1, SLC22A1, SLC22A2, Ca_v_1.2
ipratropium	9.3–9.8	9.3–9.8	9.3–9.8	9.2	8.8				ASL, OCT-2
lithocholylcholine	5.6	5.3	6.0	5.3	5.2				
mepenzolic acid		9.2	8.6	8.4					
methoctramine	6.6–7.3	7.3–8.4	6.1–6.9	6.6–7.5	6.3–7.2		5.27/6.01	GluN1/GluN2A	TRPV
methylscopolamine	9.9		10.4						
N-methylscopolamine	9.4–10.3	9.3–9.9	9.7–10.2	9.9–10.2	9.3–9.7				
ML381 (VU 0488130-1)	<5.0	<4.5	<4.5	>4.5	6.3				
MT1 toxin	7.3–7.6		7.1		<6.59			α_1_-AR	
								α_2_-AR	
MT2 toxin	6.49	4.7	4.7	6	5.7			α_1_-AR	
								α_2_-AR	
MT3 toxin (*)	7.1	<6	<6	8.5(8.7)	<6			α_2_-AR	
MT7 toxin (*)	9.8 (10.95)	<6	<6	<6	<6				
Otenzepad	5.9–6.3	6.7–7.2	6.1	6.5	5.6				
oxybutynin	8.2–8.6	7.9–8.1	8.8	8.4–8.7	7.9			DAT, σR	CYP2C19, D_3_DR, BTLP, CACNA1C, NET, 5-HT_2B_
oxyphenonium		9.75 (atria)	9.95(ileum)	9.84					α_2A_-AR
p-F-HHSiD	6.68–7.3	6.01–6.6	7.5–7.84	7.2	6.6–7.0				
PD 102807	5.3–5.5	5.7–5.9	6.2–6.7	7.3–7.4	5.2–5.5				
pirenzepine	7.8–8.5	6.3–6.7	6.7–7.1	7.1–8.1	6.2–7.1				
propantheline	9.7	9.5	10.0	10.2					
QNB	10.6–10.8	10.1–10.6	10.4	9.7–10.5	10.2–10.7			VAChT	
revefenacin	9.4	9.3	9.8	9.3	8.2				
scopolamine	9.0	8.7	9.4	9.5					5-HT_3_
SCH 57790	6.93	8.1							
silahexocyclium	8.7	7.5	8.9	8.5	8.7				
Solifenacin	7.6–8.0	6.9–7.1	7.7–8.0	6.8	7.2			K_v_11.1,	Na_v_1.5, CACNA1C, K_v_7.1, K_v_4.3
telenzepine	9.4	10.4							
	10.5 (pK_D_)								
Tiotropium	10.34	10.05	10.37	10.18	9.76				
Tolterodine	8.4–8.5	8.4–8.5	8.4–8.5	8.3–8.4	8.5–8.8			Kv11.1	Na_v_1.5, CACNA1C, Kv7.1, Kv4.3
trihexyphenidyl	8.25–8.87	7.47–7.92	7.82–8.5	8.26–9.12	7.92–8.06			σ_1_R	CYP2D6
tripinamide	7.2–7.4	7.9–9.3	5.15–5.33	6.68–6.92					
tripitramine	8.8	9.6	6.8	7.9	7.5				
tropicamide	7.08 ± 0.04	7.19 ± 0.1	6.99 ± 0.07	6.86 ± 0.12	6.42 ± 0.14				CYP2C19, CYP2C9
UH-AH 37		7.3–7.4	8.1–8.2	8.3–8.4	8.3				
umeclidinium (GSK573719)	9.8	9.8	10.2	10.3	9.9				
VU0255035	7.8	6.2	6.1	5.9	5.6				

5-HT, serotonin receptors (with specific subtypes); AANAT, serotonin N-acetyltransferase (arylalkylamine N-acetyltransferase); A_1_R, adenosine A_1_ receptor; A_2A_R, adenosine A_2A_ receptor; A_3_R, adenosine A_3_ receptor; AR, androgen receptors; ASL: argininosuccinate lyase, α_1_-AR: α_1_-adrenoceptors (with specific subtypes), α_2_-AR: α_2_-adrenoceptors (with specific subtypes), β-AR, β-adrenoceptors (with specific subtypes), BTLP, brain tumor-like proteins; CANCA1C, voltage-dependent L-type calcium channel subunit alpha-1C subunit; Ca_v_1.2, voltage-gated L-type calcium channel alpha-1C subunit; Ca^2+^/calmodulin PK II: calcium/calmodulin protein kinase II; CB R, cannabinoid receptors; CCK R, cholecystokinin receptor; cGMP-PK, cGMP dependent protein kinase; ChT, high-affinity choline transporter; CYP2C19, CYP2C9, cytochrome P450 enzymes; δ-OR, δ-opioid receptor; D_1,2,3,4,5_DR, dopamine D_1,2,3,4,5_ receptors; DAT, dopamine transporter; DAPK1, death-associated protein kinase 1; ET_A_R, endothelin ET_A_ receptors; GluN1/GluN2A subunits of NMDA glutamate receptor, GlyR, glycine receptor (subunits in parentheses); GR, glucocorticoid receptor; H_1_R, H_2_R, H_3_R, histamine receptors 1, 2, and 3; K_ir_, potassium inward rectifier; K_v_1.7, voltage-gated potassium channel 1.7; K_v_11.1, rapid delayed inward rectifying potassium current; K_v_7.1, voltage-gated potassium channel, K_v_4.3, voltage-gated potassium channel subunit K_v_4.3; LPA, Lysophospholipid receptors; MAO_A,B_, monoaminoxidase A, B; MAPK, mitogen-activated protein kinase; MC_4_R, melanocortin receptor 4; MLCK, myosin light chain kinase; MT_1A_R, melatonin receptor 1A; NMDA GluN1/GluN2A, subunits of NMDA receptors; NA, noradrenaline; Na_V_, sodium channels (batrachotoxin site); Na_v_1.5, sodium channel protein type V; OCT-2, organic cation transporter 2; TRPV, transient receptor potential vanilloid ion channel; SLC22A1, solute carrier 22, type 1; NET, norepinephrine transporter; neurotrophic rectyrK1/NGF receptor Trk-A, neurotrophic receptor tyrosine kinase 1/Nerve growth factor receptor Trk-A; NPY R, neuropeptide Y receptor; σR, sigma nonopioid receptor; SERT, serotonin (5-HT) transporter; ser/thr kinase 3, serin/threonine kinase 3; TAS2R46, TAS2R10, Taste receptor type 2 (member 46, 10); TRα, TRβ1, thyroid receptors subtypes; VAChT, vesicular acetylcholine transporter.

*pIC_50_ given instead of pK_i_. ^#^pEC_50_ given instead of pK_i_. (*): also acts as an allosteric modulator. The values in parentheses are the values for allosteric binding.

Some examples of “selective antagonists” are as follows (for the affinities of these ligands, see Table 2): pirenzepine is considered an M_1_ mAChR-selective ligand, but it binds with a similar affinity to M_4_ mAChRs (Caulfield and Birdsall, 1998; Myslivecek, 2019); methoctramine (and similarly, himbacine, AF-DX 384 and AF-DX 116) (Doods et al., 1994; Caulfield and Birdsall, 1998; Myslivecek, 2019), which is said to preferentially bind to M_2_ mAChRs, also binds with similar affinity to M_4_ mAChRs; 4-DAMP, which is known as an M_3_ mAChR-selective antagonist, reveals a similar affinity to M_4_ mAChRs (Caulfield and Birdsall, 1998; Myslivecek, 2019); and darifenacin, which is used as an M_3_ mAChR antagonist in incontinence treatment, antagonizes M_5_ mAChRs (Myslivecek, 2019). Similar non-selectivity has also been found for other mAChR antagonists. On the other hand, when working *in vitro*, some antagonists can discriminate between mAChR subtypes when a competitive binding curve is employed; for example, AFDX-384 has a significantly lower affinity for M_5_ mAChRs (pK_i_ = 6.3) than for other mAChR subtypes (pK_i_ values vary between 7.2 and 9.0). Thus, the proportion of mAChRs detected at concentrations higher than pK_i_ = 6.3 can indicate the amount of M_5_ mAChRs present [for the affinities of these ligands, see (Myslivecek, 2019)]. Telenzepine, considered M_1_ mAChR selective, has virtually the same affinity to M_1_ [pK_D_ = 10.5, (Hern et al., 2010)] as well as to M_2_ mAChRs [pKi = 10.4, (Nenasheva et al., 2013).

In the following paragraphs, we discuss the binding of mAChR antagonists to other neurotransmitter receptors and target proteins (e.g., ion channels).

Atropine, which is a typical muscarinic antagonist, can also act as an antagonist at the α_5_ subunit of NRs (although at higher concentrations, pK_i_ = 4.49) (Tomizawa and Yamamoto, 1992) and can block the effects of drugs on α_2A_- (Carr et al., 2018) and α_1D_-adrenoceptors (Gaulton et al., 2016) and glycine receptors (all subtypes (Maksay et al., 1999; Yang et al., 2007)). At higher concentrations, it also inhibits solute carrier family 22 member 1 (SLC22A1 (Ahlin et al., 2008)) and serotonin 5-HT_1A_ (Zhuang et al., 1993) and 5-HT_2C_ receptors (Gaulton et al., 2016). In mice, atropine inhibits AChE (Sowell et al., 1992) at very low concentrations (the pK_i_ is 9.15–9.46). Thus, when researchers apply atropine intending to block mAChRs, they should expect some side effects on additional receptors, which can be minor or significant. Moreover, the possible inhibition of AChE may partially artificially diminish the effects of mAChR blockade. Concerning these facts, one should consider replacing atropine with another drug (e.g., aclidinium) when trying to block mAChRs.

Similarly, the mAChR antagonist scopolamine also behaves as an antagonist of serotonin 5-HT_3_ receptors (Lochner and Thompson, 2016). Another mAChR antagonist, benzatropine, has a wider spectrum of targets including other GPCRs (D_3_ dopamine receptors, α_1A,B,D_-adrenoceptors, α_2A,B,C_-adrenoceptors (more information is available in (Svoboda et al., 2019)), H_1_ (Kulkarni et al., 2006) and H_2_ histamine receptors (Svoboda et al., 2019), and SERTs, 5-HT_2A,B,C_ and 5-HT_6_ (Modell et al., 1989)); other receptors (σR—sigma receptors (Svoboda et al., 2019)); and neurotransmitter transporters (SERT (Zhang et al., 2001), DAT (Runyon and Carroll, 2006), and NET (Newman and Kulkarni, 2002)), and it is also able to inhibit the amino acid transporter SLC6A19 (Cheng et al., 2017).

A next mAChR antagonist, methoctramine, has been proven to inhibit vanilloid receptor (transient receptor potential cation channel, TRPV) function by reducing the single-channel mean opening time and increasing the mean closure time (Mellor et al., 2004). On the other hand, it is necessary to note that the concentration used for this inhibition was relatively high (10 μmol l^−1^, i.e., 10^−5^ mol l^−1^). Another effect of methoctramine lies in GTPase activity, and it also behaves as a direct G_i_ agonist in mast cells, where it stimulates exocytosis (Chahdi et al., 1998). Additionally, it inhibits cholinesterases (AChE and BChE) (Tumiatti et al., 2003) and produces antagonistic effects on GluN1/GluN2A NMDA receptors (pK_IC50_ = 6.86, (Saiki et al., 2013)).

Amitriptyline affects mainly (in nanomolar rank) serotonin receptors (5-HT_2A_, 5-HT_2C_, 5-HT_6_, 5-HT_7_ (Gaulton et al., 2016)), H_1_R histamine receptors (Gaulton et al., 2016), α_1A,B,D_-AR, α_2A,B,C_-AR (Gaulton et al., 2016), D_1_,_2_,_3,5_DR (von Coburg et al., 2009; Gaulton et al., 2016), neurotransmitter transporters SERT, NET, and less DAT (Arunotayanun et al., 2013) and LPA1 lysophospholipid receptors (Olianas et al., 2020). Besides, potassium inward rectifiers (K_ir_3.2, K_ir_3.4) are inhibited in micromolar rank (Kobayashi et al., 2004).

Himbacine inhibits AChE, although at micromolar concentrations (Brunhofer et al., 2012). Synthetic analogs of the natural product himbacine can inhibit protease-activated receptor-1 (PAR-1), which is also known as the thrombin receptor (Chackalamannil et al., 2003; Chelliah et al., 2014). However, himbacine itself is devoid of PAR-1 activity.

Darifenacin, which has very good selectivity for M_3_ mAChRs, surprisingly affects many additional receptors and some channels and proteins. For example, darifenacine inhibits fatty acid-binding protein 4 (Wang et al., 2014), the rapid delayed inward rectifying potassium current (K_IR_, K_v_11.1), the sodium channel protein type V alpha subunit (Na_v_1.5), voltage-gated potassium channels (IKs, KCNQ1), and voltage-gated potassium channel subunit Kv4.3 (Mirams et al., 2014). Another target of darifenacin is GPCRs, including β_1_ and β_2_-adrenoceptors (Beattie et al., 2012) and dopamine D_2_ receptors (Connolly et al., 2011).

Similar to darifenacine, solifenacin affects the K_IR_, the Nav1.5/sodium channel protein type V alpha subunit, Kv7.1/IKs, KCNQ1(Kv7.1)/KCNE1(MinK), Kv4.3/IK subunit Kv4.3, and the Cav1.2/voltage-gated L-type calcium channel alpha-1C subunit (Mirams et al., 2014).

Ipratropium can activate the arginine metabolic enzyme argininosuccinate lyase (Hung et al., 2017), which is involved in specific cancer types, including hepatocellular carcinoma. Ipratropium also inhibits the organic cation transporter 2-mediated transport of specific substrates (Belzer et al., 2013).

Tropicamide is a drug usually used as an atropine replacement for pupillary dilatation. It is considered an M_4_ mAChR antagonist, but all mAChR subtypes reveal a similar affinity to this ligand (Croy et al., 2016). Moreover, this drug also inhibits the cytochrome P450 enzymes CYP2C19 and CYP2C9 (Gaulton et al., 2016). However, CYPs are affected in micromolar rank and thus they are not physiologically significant in that point of view.

Oxybutynin is another example of a drug with multiple targets. In addition to mAChRs, it also inhibits CYP2C19, D_3_ dopamine receptors, DAT (Gaulton et al., 2016), a group of brain tumor-like proteins (i.e., lethal(3)malignant brain tumor-like protein 1, L3MBTL histone methyl-lysine binding protein 3, lethal(3)malignant brain tumor-like protein 4, MBT domain-containing protein 1) (Kireev et al., 2010), voltage-dependent L-type calcium channel subunit α-1C (Wiśniowska et al., 2012), NET (Gaulton et al., 2016), SERT, 5-HT_2B_ (Gaulton et al., 2016), and sigma nonopioid intracellular receptor 1(Gaulton et al., 2016).

Tolterodine is another drug with multiple targets that binds to Kv11.1/HERG (inward rectifier) (Mirams et al., 2014), the Nav1.5/sodium channel protein type V alpha subunit (Mirams et al., 2014), the Cav1.2/voltage-gated L-type calcium channel alpha-1C subunit (Mirams et al., 2014), the Kv7.1/IK (Mirams et al., 2014), and Kv4.3/IK subunit Kv4.3 (Mirams et al., 2014).

Dicyclomine has a relatively wide spectrum of actions, in nanomolar concentrations, it inhibits 5-HT_2A_, 5-HT_2B_, 5-HT_2C_ serotonin receptors, D_3_DR, σ_1_R, σ_2_R, and in micromolar concentrations it inhibits Ca_v_1.2, H_2_R, 5-HT_6,_ Kv11.1, Na_V_ (for all data see (Gaulton et al., 2016)).

Trihexyphenidyl acts on σ_1_R in nanomolar rank and on CYPD2 in micromolar rank (Gaulton et al., 2016).

A special example of a drug with multiple targets (reveals orthosteric and allosteric binding) is MT7 toxin, which is obtained from the green mamba. This compound is not only a very selective antagonist of M_1_ mAChRs that irreversibly blocks but also binds to their allosteric sites with pK_i_ = 10.95 (Fruchart-Gaillard et al., 2006). There are further mamba toxins that bind to muscarinic receptors. MT1 toxin, however, is not specific, it binds similarly to M_1_ and M_4_ mAChRs (see Table 2), but also inhibit ^3^H-prazosin binding in different tissue preparation (in the periphery—vas deferens, in CNS—cerebral binding) indicating it is able to inhibit α_1_-ARs and α_2_-ARs (Harvey et al., 2002). It is necessary to mention that inhibition of prazosin binding was not 100% in vas deferens. Similarly, MT2 toxin is not selective and also binds to cerebral (100% inhibition) α_1_-ARs and α_2_-ARs (Harvey et al., 2002). MT3 toxin is also not selective (Jolkkonen et al., 1994; Carr et al., 2018), it also binds to the allosteric site on M_4_ mAChRs (Jolkkonen et al., 1994)and is able to inhibit α_2_-ARs (Carr et al., 2018).

Another antagonist, (S)-dimetindene is an isomer of histamine antagonist and has a similar ability to antagonize the effects on all mAChRs (Böhme et al., 2003).

Ethopropazine is also able to inhibit cholinesterases—both acetylcholinesterase and butyrylcholinesterase (Brus et al., 2014).

Very often used tritiated ligand for muscarinic receptors, ^3^H-QNB binds also to vesicular acetylcholine transporter with pK_i_ = 6.24 (Kozaka et al., 2012). However, the pK_i_ for mAChRs is around 10 (see Table 2).

Another radioligand used in mAChR detection, also as PET ligand (Mulholland et al., 1995) is tritiated, radioactively carbonylated, respectively N-methylpiperidyl benzilate (NMPB). This ligand is declared as M_2_ mAChR selective (Stein et al., 1988). However, no available data on other mAChR subtypes and structure similarity to 4-DAMP (see above) opens the question about the real selectivity of this drug. A similar case comes with clinidium, in which also data on M_2_ mAChRs are only available (Kovacs et al., 1998).

Although this review summarizes the inability of ligands to bind one mAChR subtype, this obstacle can be overcome. It is possible to use relatively specific ligands under well-defined conditions (concentration, time, and temperature), as described in KO models. Thus, pirenzepine, which is far from a selective ligand (it binds to M_1_ and M_4_ mAChRs with similar affinity), has been shown to selectively bind with a specific protocol to M_1_ mAChRs (Valuskova et al., 2018a).

### 3.2 Established antagonists of GPCRs with mAChRs as off-target with a special accent to antipsychotic drugs

Some drugs mainly act at other receptors that can antagonize the activation of mAChRs. This was described for the 5-HT_1A_ receptor agonist 7-(dipropylamino)-5,6,7,8-tetrahydronaphthalen-1-ol (8-OH-DPAT) (Fowler et al., 1991; Chidlow and Osborne, 1997). Similarly, the 5-HT_4_ receptor agonists RS 67333 and RS 67506 also reveal a low affinity to mAChRs (Eglen et al., 1995).

Special attention should be given to antipsychotics. These drugs are a perfect example of multitargeting advantage in treating specific diseases. All drugs listed here can bind not only to “classical” antipsychotic targets like serotonin, dopamine, or histamine receptors but also inhibit muscarinic receptors which are additional, desirable effects that could widen the action of antipsychotics. This is valid for the following drugs. Haloperidol, a typical antipsychotic medication, in addition to its classical effects on dopamine and serotonin receptors, is also able to inhibit the M_2_ mAChRs, although the affinity is not too high (pK_i_ = 6.62, (Ronsisvalle et al., 1998)). Olanzapine, which is an atypical antipsychotic with a higher affinity for 5-HT_2A_, SERTs, and D_2_ dopamine receptors, has also been shown to antagonize the effects of pilocarpine on phosphoinositide hydrolysis, although with lower affinity (Zhang and Bymaster, 1999). In general, many drugs used as antipsychotics (usually targeting dopamine and serotonin receptors), such as chlorpromazine, fluphenazine, levomepromazine, perphenazine [for all these drugs see (Hals et al., 1988)], and blonanserin (Tenjin et al., 2013) have anticholinergic effects and can antagonize the binding of mAChRs. In the first generation of antipsychotics, thioridazine reveals a wide spectrum of effects which also include muscarinic (M_1_-M_5_) antagonism in the range pK_i_ = 6-10 (Vass et al., 2018). Clozapine is one of the first atypical antipsychotics. Besides its antagonistic effects on histamine, serotonin, adrenergic, and dopamine receptors, it also reveals positive allosteric effects on M_1_ mAChRs (Flamez et al., 1994) with high affinity (pK_IC50_ = 7.9 (Sur et al., 2003), antagonistic effects on M_2_ mAChRs pK_i_ = 7.32–7.36 (Ohmori et al., 1996), and on M_3_-M_5_ mAChRs (Bandyopadhyaya et al., 2012) with pK_i_ = 7-8). This also led to the possibility that muscarinic receptors can be targeted when treating schizophrenia (Foster et al., 2021). In this review, clozapine, xanomeline, and specific allosteric modulators targeting to mAChR subtypes (MK-801 (M_4_ mAChR selective), BQCA (M_1_ mAChR selective), VU0238429 (M_5_ mAChR selective) are discussed. It is necessary to mention that there can also be a possibility that xanomeline act on dopaminergic neurons through its action on M_1_ and M_4_ mAChRs (Weiden et al., 2022). Similarly, the effects of MK-801, BQCA, and VU0238429 are the most probably via the mAChRs expressed on dopaminergic/serotoninergic or other neurons (Foster et al., 2021). An important aspect of antipsychotic action has been investigated by (Obara et al., 2019) who determined the inhibitory effects on brain mAChRs in therapeutically achievable concentrations. They found that the pK_i_ values of chlorpromazine (pK_i_ = 5.85–7.55), levomepromazine (pK_i_ = 6.21–7.35), and zotepine (pK_i_ = 6.14–7.04), olanzapine (pK_i_ = 6.37–7.38), and clozapine (pK_i_ = 5.23–6.30) overlapped with their clinically achievable blood concentrations. Importantly, clozapine act as a positive allosteric modulator (see Table 3) with pK_IC50_ = 7.7–7.9 at M_1_ mAChRs (Sur et al., 2003; Spalding et al., 2006) and together with this action it also competes with M_1_ mAChRs [pK_i_ = 7.0–9.01 (Rowley et al., 2001; Bandyopadhyaya et al., 2012)], with M_2_ mAChRs [pK_i_ = 6.77–7.34 (Gaulton et al., 2016)], with M_3_ mAChRs [pK_i_ = 7.0–7.77 (Bandyopadhyaya et al., 2012; Gaulton et al., 2016)], with M_4_ mAChRs [pK_i_ = 7.0–8.2 (Bandyopadhyaya et al., 2012; Gaulton et al., 2016)], and with M_5_ mAChRs [pK_i_ = 7.0–8.02 (Bandyopadhyaya et al., 2012; Gaulton et al., 2016)]. Another antipsychotic, dosulepin, act with high affinity on histamine receptors (Sánchez and Hyttel, 1999), SERT, and NET(Tatsumi et al., 1997) and, of course, on all mAChR subtypes (Stanton et al., 1993). Thus, it is possible to conclude that mAChRs play important role in antipsychotic action, arising from the effects on mAChR subtypes that are integral to the regulation of DA neural circuits (Weiden et al., 2022).

**TABLE 3:** Effects of allosteric ligands on muscarinic receptors and other targets. The compounds are listed alphabetically. The numbers indicate pKi values. Please note that a higher pKi indicates higher affinity. Unavailable data are shown as blank spaces. The affinities of nicotinic receptors are not divided among specific subunits. The affinities of other, noncholinergic targets are divided into nanomolar (pKi>6) and micromolar (pKi<6) groups. The categorization of pKi is compromise between extremely large Table and fast orientation how much are other targets are affected in specific concentration of ligand used (see also examples in Chapter 5). Data are from or adapted from (Pascuzzo et al., 1984; Eglen and Whiting, 1987; Buckley et al., 1989; Buckley et al., 1990; Hudkins et al., 1991; Wess et al., 1991; Bolden et al., 1992; Kashihara et al., 1992; Sowell et al., 1992; Doods et al., 1993; Shen et al., 1993; Stanton et al., 1993; Doods et al., 1994; Ferrari-Dileo et al., 1994; Kleinschroth et al., 1995; Waelbroeck et al., 1996; McKenna et al., 1997; Boess et al., 1998; Bolognesi et al., 1998; Cantí et al., 1998; Caulfield and Birdsall, 1998; Lazareno et al., 1998; Choppin et al., 1999; Sánchez and Hyttel, 1999; Eglen and Nahorski, 2000; Kozlowski et al., 2000; Lazareno et al., 2000; Becerra et al., 2001; Dhein et al., 2001; Huang et al., 2001; Lockhart et al., 2001; Apelt et al., 2002; Carlsson et al., 2002; Harvey et al., 2002; Cheng et al., 2002; Lazareno et al., 2002; Böhme et al., 2003; Samochocki et al., 2003; Sur et al., 2003; Jakubik et al., 2004; Kobayashi et al., 2004; Wang et al., 2004; Ghoneim et al., 2006; Spalding et al., 2006; Butini et al., 2008; Langmead et al., 2008; Bridges et al., 2009; Heinrich et al., 2009; Prat et al., 2009; von Coburg et al., 2009; Bridges et al., 2010a; Harada et al., 2010; Hern et al., 2010; Lange et al., 2010; Rook et al., 2010; Sinha et al., 2010; Watt et al., 2011; Daval et al., 2012; Samadi et al., 2012; Sykes et al., 2012; Arunotayanun et al., 2013; Nenasheva et al., 2013; Salmon et al., 2013; Croy et al., 2016; Gaulton et al., 2016; Alexander S. P. et al., 2017; Alexander S. P. H. et al., 2017; Chen et al., 2017; Carr et al., 2018; Hegde et al., 2018; Broad et al., 2019; Myslivecek, 2019; Xu et al., 2019; Olianas et al., 2020; Okimoto et al., 2021) and the IUPHAR/BPS Guide to Pharmacology (www.guidetophamacology.org). For specific ligand references see text. The activity represents the main effect of a specific ligand.

Target	Muscarinic receptors					Nicotinic receptors	Cholinester-ases (ChEs) AChE/BChE	Other targets	
	M_1_	M_2_	M_3_	M_4_	M_5_			Nanomolar (pKi > 6)	Micromolar (pKi < 6)
AC-260584	(5.9)P^*^	5.0(An)	5.23(An)	5.0(An)	6.0(Ag)				
ASP8302			P (20.1 fold increase with 0.3 µmol/L)		P (22.1 fold increase with 0.3 µmol/L)				
Alcuronium	(5.0)N	(6.1–6.9)N	(5.8)N	(5.6)N		7.3			
Amiodarone			(5.5–6.3)		(7.3)			σR	Nav1.5, K_v_1.7, β_1,3_-AR, α_2A,B_-AR, AR, D_1-4_DR, 5-HT_1A_, HT_2A,B,C_, 5-HT_6_, TRα, TRβ1
Brucine	(4.5–5.8) P	(4.3–4.6) P	(3.6–4.0) N	(4.7–6.0) N	(2.9) N				
	(4.5) Neu	(4.3) N							
brucine N-oxid	(3.2) P,Neu	(3.5) N,P	(2.5) P, Neu	(3.6) P, Neu	(3.3) N,P				
Clozapine	7.0–9.01 (*)	6.77–7.34	7.0–7.77	7.0–8.2	7.0–8.02			5-HT_1A,B,D,e,F_ 5-HT_2A,B,C_ 5-HT_5A_ 5-HT_6_, 5-HT_7_ SERT D_1-5_ DR H_1,4_ R α_1A,B,D_-AR K_v_11.1/HERG	K_ir_3.2
	(7.7–7.9)*P								
Gallamine	5.07	(5.6-7-6)				6.0	4.43–4.82		
Gö 7874	(5.8)N	(5.0)N	(5.1)N	(5.7)Neu					cGMP-PK, MLCK
K-252a	(5.1)P							Ca^2+^/calmo-dulin PK II MAPK 9 MAPK 10 MAPK 11 MLCK, cGMP-PK,neurotrophic rectyrK1/NGF receptor Trk-A	
KT5720	(6.4)P		(6.4)N					PKA	
KT 5823	(5.7)P	(5.7)P							
Lu AE51090	(7.2) Pa	5.66	5.15	5.16	5.05		6.0	α_1A,B_-AR	
								H_1_ R	
LY20332298		(6.0) P N		(6.7)					
LY2119620		(5.5–5.7) P, Pa		(5.5) P, Pa					
McNeil-A-343^$$^	4.8–5.2	4.7–6.0	5.0–5.3	5.6–6.7	4.9				5-HT_3_, 5-HT_4_
MK-7622	(7.0)P								arachidonate 5-lipoxygenase, Kv11.1/HERG
MT3 toxin	7.1	<6	<6	8.5 (8.7)	<6			α_2A_-AR	
MT7 toxin	9.8 (10.95)	<6	<6	<6	<6				
N-benzyl-brucine	(4.4)N	(4.8)N,P	(3.8)N,P	(4.5)N,Neu	(3.7)N,Neu				
N-chloromethyl-brucine	(4.1)N	(4.6)N,P	(3.3)P	(4.4)Neu	(4.4)N				
N-desmethylclozapine	(6.8–7.3)P							5-HT_1A_, δ-OR, 5-HT_6_	5-HT_2C_
staurosporine	(5.9)P	(5.1)P		(5.3)Neu				Ca^2+^/calmodulin PK II, ser/thr kinase 3, DAPK1	
Strychnine	(4.9–5.0) Neu, N	(4.9–5.0)P	(4.2–5.7)N	(4.8–5.0)P	(3.6)N			GlyR (α_1_, β, α_2_, α_3_),	TAS2R10
								TAS2R46	
Tacrine	(5.7)N	(5.7)N				6.82	7.5/7.2		α_1Α_-AR, CB_1_, CB_2_, GluN1/GluN2A, MAO_A_, MAO_B_, histamine N-methyltransfer-ase, SERT, SLC22A1
thiochrome	(4.1)Neu	(3.9)Neu	(4.4)Neu	(4.0)P					
Vinburnine	(5.1)Neu	(4.2)Neu	(5.2)Neu	(4.6)P					
Vincamine	(4.8)Neu	(5.1)Neu	(5.7)Neu	(4.2)P					
VU0119498	(5.2)P	(4.52)P	(5.2)P	(4.52)P	(5.4)P				
W-84		(6.0–7.5)P (7.6)N							
WIN 51,708	(5.8)N	(5.9)N	(5.5)N	(6.2)N					
WIN 62,577	(5.5)N	(5.3)N	(5.1)P	(5.9)N				NK1	

5-HT, serotonin receptors (with specific subtypes); AANAT, serotonin N-acetyltransferase (arylalkylamine N-acetyltransferase); A_1_R, adenosine A_1_ receptor; A_2A_R, adenosine A_2A_ receptor; A_3_R, adenosine A_3_ receptor; AR, androgen receptors; ASL, argininosuccinate lyase; α_1_-AR, α_1_-adrenoceptors (with specific subtypes); α_2_-AR, α_2_-adrenoceptors (with specific subtypes); β-AR, β-adrenoceptors (with specific subtypes); BTLP, brain tumor-like proteins; CANCA1C, voltage-dependent L-type calcium channel subunit alpha-1C subunit; Ca_v_1.2, voltage-gated L-type calcium channel alpha-1C subunit; Ca^2+^/calmodulin PK II, calcium/calmodulin protein kinase II; CB R, cannabinoid receptors; CCK R, cholecystokinin receptor; cGMP-PK, cGMP dependent protein kinase; ChT, high-affinity choline transporter; CYP2C19, CYP2C9, cytochrome P450 enzymes; δ-OR, δ-opioid receptor; D_1,2,3,4,5_DR, dopamine D_1,2,3,4,5_ receptors; DAT, dopamine transporter; DAPK1, death-associated protein kinase 1; ET_A_R, endothelin ET_A_ receptors; GluN1/GluN2A subunits of NMDA glutamate receptor, GlyR: glycine receptor (subunits in parentheses), GR: glucocorticoid receptor, H_1_R, H_2_R, H_3_R: histamine receptors 1, 2, and 3, K_ir_: potassium inward rectifier; K_v_1.7, voltage-gated potassium channel 1.7; K_v_11.1, rapid delayed inward rectifying potassium current; K_v_7.1, voltage-gated potassium channel; K_v_4.3, voltage-gated potassium channel subunit K_v_4.3; LPA, Lysophospholipid receptors; MAO_A,B_, monoaminoxidase A, B; MAPK, mitogen-activated protein kinase; MC_4_R, melanocortin receptor 4; MLCK, myosin light chain kinase; MT_1A_R, melatonin receptor 1A; NMDA GluN1/GluN2A, subunits of NMDA receptors; NA, noradrenaline; Na_V_, sodium channels (batrachotoxin site); Na_v_1.5, sodium channel protein type V; OCT-2, organic cation transporter 2; TRPV, transient receptor potential vanilloid ion channel; SLC22A1, solute carrier 22, type 1; NET, norepinephrine transporter, neurotrophic rectyrK1/NGF receptor Trk-A, neurotrophic receptor tyrosine kinase 1/Nerve growth factor receptor Trk-A; NPY R, neuropeptide Y receptor; σR, sigma nonopioid receptor; SERT, serotonin (5-HT) transporter; ser/thr kinase 3, serin/threonine kinase 3; TAS2R46, TAS2R10, Taste receptor type 2 (member 46, 10), TRα; TRβ1, thyroid receptors subtypes; VAChT, vesicular acetylcholine transporter.

(Ag) agonist. (An) antagonist. (*): also acts as an allosteric modulator. The values in parentheses are the values for allosteric binding. The values in brackets are the values when ligand also act as partial agonist. P, PAM; N, NAM; Neu, neutral allosteric modulator; F, full allosteric agonist; Pa, partial allosteric agonist (shown when dual effects are present; otherwise, see the text).

Imipramine, a tricyclic antidepressant, has a wide range of targets: besides M_2_ (Kovacs et al., 1998) and M_5_ mAChRs (Griffith et al., 1984), it binds also to α_1A_-AR (Yardley et al., 1990), D_2_ DR (Peters, 2013), H_1_ R (Peters, 2013), 5-HT_2A,C_ (Peddi et al., 2004), NET, and SERT (Runyon et al., 2001).

Similar multitarget effects can be found (Vass et al., 2018) for astemizole, an H_1_ histamine receptor antagonist, and cinnarizine was used primarily as an anti-histaminic. All these three drugs were withdrawn from the market because of their side effects. Similarly, econazole - an antifungal agent, primarily acting on TRPM and TRPV channels (Hill et al., 2004) has also anti-muscarinic effects (M_1_, M_3_, M_4_) (Vass et al., 2018).

One should keep it in mind when applying antipsychotics in an experimental condition. As it can be seen one also should be cautious when limiting the effects of antipsychotics on serotonin, dopamine, and histamine receptors only.

An interesting aspect of interneurotransmitter effects has recently been shown—the simple replacement of a flexible alkyl chain with a semirigid aryl or cycloalkyl ring leads to increased mAChR affinity of specific ligands, detrimentally affecting histamine receptor binding (Staszewski et al., 2021). However, these newly synthesized ligands continued to bind to histamine receptors, although with lower affinity.

## 4 Muscarinic receptor allosteric ligands

The allostery concept was introduced in 1961 by Monod and Jacob and suggests conformational changes in a two-state model [see (Coughlin et al., 2019)]. The search for allosteric mAChRs ligands began in the 1970s (Clark and Mitchelson, 1976) and was further developed in the 1980s.

The general principle of allosteric action lies in the fact that binding to allosteric sites can modulate the activity of orthosteric ligands (Kruse et al., 2014). The common mechanisms of allosteric modulation have been indicated to be generalizable and can be found for many if not all, GPCRs. However, marked structural differences also exist. Mainly among mAChR subtypes, the receptors reveal different sequence homology. While orthosteric site sequence homology is high between all mAChR subtypes, in the extracellular parts of the transmembrane domains and extracellular loops of these receptors, there is a low degree of sequence homology. In summary, the interactions of ligands with receptors can occur as follows (Mohr et al., 2013): 1) positive allosteric modulators (PAMs) increase the affinity or efficacy of an orthosteric ligand or orthosteric agonist–receptor complex, increase the equilibrium binding of the simultaneously bound endogenous or exogenous orthosteric ligand, enhance either the affinity of an orthosteric agonist or lower the activation barrier for the transition from the inactive to the active conformation of the receptor, 2) negative allosteric modulators (NAMs) decrease the affinity or efficacy of an orthosteric ligand or orthosteric agonist–receptor complex, inducing a rightward shift of the concentration-effect curve of the endogenous agonist (as an example), 3) neutral allosteric ligands (NALs) bind to an allosteric site but have no effect on the affinity or efficacy of orthosteric or allosteric ligands, and 4) allosteric agonists activate the receptors themselves.

We can also define some strengths and disadvantages of each mode of mAChR targeting. Orthosteric sites exhibit high affinity to ligands, but mAChR subtype selectivity remains largely unfeasible. Allosteric sites exhibit greater receptor selectivity and promote more physiological response patterns, but there was a problem with low-affinity modulators that have been reported (Melancon et al., 2012b). Recently, some drugs that overcame this obstacle advanced to the preclinical or clinical phase of testing in the treatment of neurological disorders (Moran et al., 2019).

The possible solution to this problem could be bitopic (dualsteric) ligands. Research has been conducted to rationally design hybrid molecules that simultaneously bridge orthosteric and allosteric sites within a single receptor. These compounds have been termed “bitopic,” “dualsteric,” or “multivalent.” These drugs attempt to target allosteric sites to achieve selectivity and orthosteric sites to provide high affinity (Bock et al., 2018).

At the beginning of the 21st century (Disingrini et al., 2006), first combined a nonselective, high-affinity orthosteric agonist (iperoxo) with an M_2_ receptor-selective allosteric modulator to generate an M_2_ receptor-selective agonist. However, although bitopic agonists with mAChR subtype selectivity and signaling tendencies have been described, substantial improvements in affinity were not observed [see (Kruse et al., 2014)]. On the other hand, the bitopic mAChR antagonist THRX-160209 has been shown to exhibit both affinity and selectivity for the M_2_ receptor (Steinfeld et al., 2007b).

The selective ligands (the current situation) are summarized in Table 4. It is necessary to stress that very well-established ligands (with a long history of use in mAChR research, e.g., atropine) are expected to have more additional targets than less established ligands (e.g., allosteric ligands with a short history of use or only recently discovered), simply because they are better investigated. Thus, one should consider Table 4 as the present state of knowledge.

**TABLE 4:** The list of selective MR ligands. The present state of knowledge. The values in parentheses represent pK_i_ (pK_D_, respectively) for M_1_, M_2_, M_3_, M_4_, and M_5_ MRs). Some orthosteric agonists ((-)YM796, AZD6088, LSN3172176, butylthio-TZTP are not sufficiently documented with respect to selectivity. The same can be stated about allosteric agonist listed in this table (ML169, PF-06767832, T-495) that have been reported as selective but the data on other MR subtypes are missing. VU0029767 and VU0090157 showed selectivity on M_1_ MRs over other subtypes but values were not reported.

Muscarinic subtypes					
M1	M_2_	M_3_		M_4_	M_5_
Agonist	benzoquinazolinone 12*P	dimethyl-W84*P		VU0010010*P	**ML380** ^$^P
	(6.55)	(8.5)		(6.4) ^$^	(5.25, 4.5, 5.6, 4.5, 6.72)
	Abdul-Ridha et al. (2014)	Tränkle et al. (2003)		Shirey et al. (2008)	Gentry et al. (2014b)
	**BQCA** P,F	Duo3*P		VU0152099*P	**VU0238429** (ML 129)^$^P
	(4.0–4.8, all other subtypes are not affected by 10^−4^ mol/L BQCA)	(7.1)		(6.4)^$^	(4.52, 4.52, 4.52, 4.52, 5.96)
	Ma et al. (2009)	Tränkle et al. (2005)		Brady et al. (2008)	Gentry et al. (2013a)
	ML169*P	WDuo3*P		VU0152100*P	**ML326** (VU0467903)^$^P
	(5.9)	(6.9)		(6.64)^$^	(<4.52, <4.52, <4.52, <4.52, 6.39)
	Tarr et al. (2012)	Tränkle et al. (2003)		Le et al. (2013b)	Gentry et al. (2013a)
	PF-06767832*			**compound-110** ^#^	**VU0400265** ^$^ P
	(7.2)P			Wang et al. (2022)	(<4.52<4.52, <4.52, <4.52, 5.72)
	Davoren et al. (2016)				Bridges et al. (2010b)
	T-495*P			**compound 24** ^#^	**VU0365114** ^$^ P
	(6.19)			Schubert et al. (2019)	(<4.52<4.52, <4.52, <4.52, 5.56)
	Mandai et al. (2020)				Bridges et al. (2010b)
	**VU0029767** ^#^P			**VU0467154** ^$^ P	
	Marlo et al. (2009)			(NA,NA,NA, 7.75, NA)	
				Bubser et al. (2014)	
	**VU0090157** ^#^P			**ML293** ^$^*P	
	Marlo et al. (2009)			(5.89)	
				Salovich et al. (2012)	
	AZD6088^$^			**ML253** ^$^*P	
	(8.3, 6.82, <6,<6,<6)			(7.25)	
	(MRC/AstraZeneca: Mechanisms of Disease)			Le et al. (2013a)	
**Antagonist**	VU0255035	N-methylpiperidyl benzilate (NMPB)*		PCS1055^#^	**ML381** (VU 0488130-1) N
	(7.8, 6.2, 6.1, 5.9, 5.6)	(9.4)			(<5, <5, 4.5, >4.5, 6.3)
	Sheffler et al. (2009)	Stein et al. (1988)		Croy et al. (2016)	Gentry et al. (2014a)
	MT7 toxin (*)				**ML375** ^$^ N
	(9.8 [10.95], <6, <6, <6, <6)				(<4.52, <4.52, <4.52, <4.52, 6.52)
	Caulfield and Birdsall, (1998)				Gentry et al. (2013b)
	quinuclidinyl-4-fluoromethyl-benzilate^#^	dexetimide* (8.9)			
	Kiesewetter et al. (1997)	Kovacs et al. (1998)			
		levetimide*			
		(5.0)			
		Kovacs et al. (1998)			
		**THRX160209** N (*)			
		(≤8.0, 9.5, ≤8.0, 8.78, ≤8.0)			
		Steinfeld et al. (2007a)			
		**C7/3-phth** N*			
		(7.1)			
		Avlani et al. (2007)			

Orthosteric ligands are shown in normal typeface, allosteric ligands are shown in bold typeface. *value for only one MR subtype was referred. ^#^values not given. ^$^pEC_50_ given instead of pK_i_. ^$$^pEC_50_ given instead of pK_i_. (*): orthosteric binding, but also acts as an allosteric modulator (value in brackets is pK_i_ for allosteric action). P, PAM; N, NAM; Neu, neutral allosteric modulator; F, full allosteric agonist; Pa, partial allosteric agonist (shown when dual effects are present; otherwise, see the text). NA, no action; i.e. no effect on the receptor.

Despite their relatively short history of use, some allosteric ligands have also been shown to target multiple receptors. Following are examples of such allosteric (or bitopic) ligands.

Some of these drugs can act not only on mAChRs but also on other neurotransmitter receptors. This is the case for McNeil-A-343, which acts on all mAChR subtypes as a partial agonist (Alexander S. P. et al., 2017). In addition, it is also considered a bitopic ligand of mAChRs in that it modulates allosteric and orthosteric binding sites (Christopoulos et al., 2014). In addition, this compound can also act as an antagonist of 5-HT_4_ and 5-HT_3_ receptors at a range of concentrations overlapping with those relevant to its interaction with mAChRs (Sagrada et al., 1994). An example of a compound with unprecedented selectivity and procognitive potential is Lu AE51090, an allosteric muscarinic M_1_ mAChR agonist. Although the selectivity to M_1_ mAChR is really nice (between 36-145× better than to M_2_-M_5_ mAChRs), there is still binding to α_1A,B_-adrenoceptors, and to histamine H_1_ receptors (pK_i_s between 6.07-6.59, (Sams et al., 2010)). Thus, this compound shows the advantage of multitarget binding in treatment.

Another example of mAChR allosteric ligands acting on other neurotransmitter receptors is as follows. MT3 toxin from the green mamba is considered an M_4_ mAChR NAM. However, it has also been demonstrated to be an α_2A_-adrenoceptor antagonist (Carr et al., 2018). The opposite mAChR allosteric ligand situation is seen in amiodarone, which is a classical blocker of voltage-gated potassium channels (K_v_1.7) (Bardien-Kruger et al., 2002) and voltage-gated sodium channels (Na_v_1.5)(Sheldon et al., 1989). Relatively recently, it has also been identified as a PAM of M_5_ mAChRs (Stahl and Ellis, 2010), but it has an alternative allosteric binding site (Burger et al., 2021). This drug also acts as an M_3_ mAChR PAM (Stahl et al., 2011). However, this molecule has additional targets, including β-adrenoceptors (Chatelain et al., 1995), thyroid hormone receptors α and β (Carlsson et al., 2002), and other molecules [α_2_-adrenoceptors, dopamine receptors, 5-HT receptors, and σ nonopioid receptors; see (Gaulton et al., 2016)].

The first pioneer molecules with described allosteric effects on mAChRs were originally targeted toward NRs. This is the case for alcuronium, which is a neuromuscular-blocking drug with negative allosteric effects on mAChRs (Nedoma et al., 1985) that acts as a NAM of M_1_, M_2_, M_3,_ and M_4_ mAChRs. Gallamine (defined as a nondepolarizing muscle relaxant) is a NAM of M_2_ mAChRs and has a similar affinity to the α_1_ subunits of NRs, which are inhibited by this drug [e.g., (Psaridi-Linardaki et al., 2003)]. Higher concentrations of gallamine can also inhibit AChE (Butini et al., 2008). Gallamine also exhibits competitive inhibition of M_1_ mAChR (Daval et al., 2012). Tacrine was first synthesized as a dual inhibitor of butyrylcholine esterase and acetylcholinesterase with the aim to treat Alzheimer’s disease (Fisher, 2008). In addition to these effects (both cholinesterases have a relatively high affinity to tacrine with pK_i_ ≈ 7), this drug also reveals negative allosterism on M_1_ and M_2_ mAChRs [pIC_50_ = 5.7, (Sowell et al., 1992)]. Tacrine also inhibits nicotinic receptor subunit ε (Xu et al., 2019), at a similar concentration as for mAChRs it inhibits α_1Α_-adrenoceptors (Gaulton et al., 2016), cannabinoid receptors CB_1_, CB_2_ (Lange et al., 2010), GluN1/GluN2A/Glutamate NMDA receptor subunits (Rook et al., 2010), histamine N-methyltransferase (Apelt et al., 2002), MAO_A_ and MAO_B_ (Samadi et al., 2012), SERT (McKenna et al., 1997), and SLC22A1 (Chen et al., 2017).

Staurosporine, as another example of a drug with a classical function, is described mainly as a protein kinase inhibitor but is also a PAM of M_1_ and M_2_ mAChRs (Lazareno et al., 2000). In addition, it can affect many other molecules, such as calcium calmodulin protein kinase II, serine/threonine kinase 3, and death-associated protein kinase 1 [see (Hall et al., 2009)]. Brucine is a natural alkaloid product with many effects on organisms (Lu et al., 2020), such as antitumor, anti-inflammatory, analgesic effects, and effects on the cardiovascular system and nervous system. This molecule is also a PAM of M_1_ and M_2_ mAChRs and a NAM of M_3_, M_4,_ and M_5_ mAChRs (Jakubík et al., 1997; Lazareno et al., 1998; Birdsall et al., 1999). WIN 62,577, as another allosteric modulator, is a NAM of M_1_, M_2_, M_4,_ and PAM of M_3_ mAChR that was originally synthesized as an NK1 antagonist (Lazareno et al., 2002). Another allosteric ligand, AC-260584 has also complicated action: it acts as PAM on M_1_ mAChR (pIC_50_ = 5.9) and other mAChR subtypes (M_2_-M_4_) reveal similar affinity when it acts as a full antagonist. On M_5_ mAChR it has agonist action and the affinity is also similar (Melancon et al., 2012a). Strychnine, glycine antagonist (Gaulton et al., 2016)—with action on many subunits (Zlotos et al., 2019), can allosterically modulate the muscarinic transmission: positively, negatively, as well as it has a neutral effect on mAChRs (Jakubík et al., 1997) also affects taste receptors (Gaulton et al., 2016).

Another M_1_ and M_4_ mAChR PAM, KT5720 (Lazareno et al., 2000), is also a protein kinase A inhibitor (Birdsall et al., 2001). Kinases (cGMP-dependent protein kinase and myosin light chain kinase) are inhibited in mAChRs (M_1_-M_4_) action rank by Gö 7874 (Kleinschroth et al., 1995; Lazareno et al., 2000). Another positive allosteric modulator, K-252a, selective to M_1_ mAChRs (Lazareno et al., 2000), is, however, a potent inhibitor of many kinases in rank (pKi (pIC_50_, respectively, between 6.44 and 8.74) above the action on M_1_ mAChRs (pK_d_ = 5.1)—see Table 3. N-desmethylclozapine is a specific M_1_ mAChR PAM but also has the properties of an atypical agonist of M_1_-M_4_ mAChRs (Sur et al., 2003). In addition, N-desmethylclozapine is a potent 5-HT_1A_ (Heusler et al., 2011) partial receptor antagonist, an antagonist of 5-HT_2C_ receptors (Hasuo et al., 2002) and cloned 5-HT_6_ receptors (Dupuis et al., 2008) and an agonist of δ-opioid receptors (Olianas et al., 2009).

An example of a selective drug (PAM) for M_1_ mAChRs is MK-7622. The pEC_50_ = 7.0 (Bertron et al., 2018) seems to be far from the pIC_50_s (see (Beshore et al., 2018)) for arachidonate 5-lipoxygenase (pIC_50_ = 5.36) and Kv11.1/HERG (pIC_50_ = 4.22). With respect to the common criteria for receptor selectivity, it looks good, and one could consider MK-7622 as an M_1_ selective ligand. This is true but a cautious attitude is recommended concerning possible future discoveries (the drug showing the selectivity at present would be less investigated than notoriously known drugs).

Other examples present the situation in which the activities of ligands depend on the number of mAChRs present in the tissue in which the agonist is used for receptor activation. For example, the M_1_ mAChR selective modulator BQCA acts solely as a PAM of ACh activity when assayed in a cell line with low M_1_ mAChR density (Canals et al., 2012), and although signaling pathways are weakly coupled to the M_1_ mAChR, it acts as both a full allosteric agonist and a PAM in a system with a high M_1_ mAChR reserve. Another example of such a ligand is LY2033298, which is usually considered an M_4_-selective agonist and has a more complicated mode of action. It also binds to M_2_ mAChRs (Valant et al., 2012) and mediates both positive and negative allosteric effects, depending on the orthosteric ligand. More concretely, it is a PAM of oxotremorine-M signaling at M_2_ mAChRs but a NAM of xanomeline at the same receptor (Valant et al., 2012). Another ligand with mixed pharmacodynamics is LY2119620. LY2119620 acts as a PAM of ACh on M_2_ mAChRs and M_4_ mAChRs but also exhibits partial allosteric agonism of M_2_ and M_4_ mAChRs (Croy et al., 2014; Schober et al., 2014). Dual effects are seen in W-84 which act both as PAM and NAM on M_2_ mAChRs (Tränkle et al., 2003). Relatively recently, the allosteric modulator of M_3_ mAChR was described and introduced in the clinic for the treatment of underactive bladder (Okimoto et al., 2021). This drug acts as PAM, potentiate activation, not only on M_3_ mAChR but with similar effects on M_5_ mAChR.

Another characteristic of GPCR allostery is biased agonism, which refers to the abilities of different ligands to stabilize a subset of functionally relevant GPCR conformations, such that different signaling outputs are achieved with the exclusion of others (see (Kruse et al., 2014)). An example of this type of ligand is the compound VU0029767, which potentiates ACh-mediated phospholipase C activity via the M_1_ mAChR. ACh-induced phospholipase D activation is not, however, affected (Marlo et al., 2009).

Similarly, the development of multitarget drugs with anti-psychotic effects (antagonists on D_2_ dopamine receptors and 5-HT_2A_ serotonin receptors) with allosteric agonism is a promising strategy how to minimize the side effects of drugs (Szabo et al., 2015).

## 5 Nonsimple targeting of muscarinic drugs

In this section, three examples of experiments in which orthosteric mAChR agonists/antagonists were used are described. These papers were selected to demonstrate not only the need for selectivity awareness but also the role of the mAChR subtype ratio in the respective tissue. This section aims to show the trickiness of muscarinic ligand use and possible ways to avoid misinterpretations of obtained data. The reader can thereby be aware of the problems associated with mAChR agonist and antagonist specificity.

As an example (Morales-Weil et al., 2020), used different muscarinic antagonists to determine the subtype responsible for long-term GABAergic (inhibitory) potentiation. In a conclusion from their experiments, they declared that M_1_ mAChRs are responsible for the blockade of long-term inhibitory potentiation at GABA synapses in the hippocampus. The authors used pirenzepine at a concentration (100 nmol l^−1^, i.e., 10^−7^mol l^−1^) with which it was not possible to exclude the blockade of not only M_1_ mAChRs but also other mAChR subtypes, at least in part. Considering the pK_i_s of pirenzepine for specific mAChR subtypes (Myslivecek, 2019) (M_1_: pK_i_ = 7.8–8.5, M_2_: pK_i_ = 6.3–6.7, M_3_: pK_i_ = 6.7–7.1, M_4_: pK_i_ = 7.1–8.1, and M_5_: pK_i_ = 6.2–7.1), it is evident that at 10^−7^ mol l^−1,^ pirenzepine would block the majority of M_1_ mAChRs and the majority of M_4_ mAChRs. We can also assume that approximately one-half of M_3_ mAChRs were occupied by pirenzepine (pK_i_ = 6.7–7.1), that M_5_ mAChRs were probably occupied in the same way, and that M_2_ mAChRs were also partly occupied. Under this condition, focal ACh induced short-term depression of GABAergic transmission, which led to the suggestion that presynaptic M_2_ mAChRs are responsible for this phenomenon. The authors also used AFDX-116 (10 μmol l^−1^, i.e., 10^−5^ mol l^−1^) as an M_2_ mAChR antagonist and found no effects of this antagonist on long-term inhibitory potentiation, concluding that M_2_ mAChRs are not involved in this form of long-term plasticity. However, upon comparing the pK_i_s of AFDX-116 for specific mAChR subtypes (Myslivecek, 2019) (M_1_: pK_i_ = 5.8–6.9, M_2_: pK_i_ = 7.1–7.3, M_3_: pK_i_ = 5.5–6.6, M_4_: pK_i_ = 6.2–7.0, and M_5_: pK_i_ = 5.4–6.6), it is apparent that not only M_2_ mAChRs but also M_4_ mAChRs and some M_1_, M_3_ and M_5_ mAChRs were inhibited by 10^−5^ mol l^−1^ AFDX-116. However, considering that the relative density of M_1_ mAChRs in the whole hippocampus ranges between 69% (Oki et al., 2005) and 91% (Valuskova et al., 2018a) and is approximately 89% in the CA1 hippocampal area (Valuskova et al., 2018a) and that separate studies indicated that M_2_ and M_4_ mAChRs were undetectable/insignificant in the hippocampus and CA1 area (Valuskova et al., 2018a) or that M_2_ mAChRs represented 31% of receptors (Oki et al., 2005) while M_4_ mAChRs were undetectable (Oki et al., 2005; Valuskova et al., 2018a), it is possible to conclude that the role of M_1_ mAChRs is indisputable and that M_2_ mAChRs are not highly present in hippocampal areas. In summary, it is necessary to consider not only the limited selectivity of mAChR antagonists but also the relative presence (or subtype ratios) of the respective mAChR subtypes.

Another aspect that can lead to problems in results interpretations is the *in situ* hybridization of mAChR subtype nucleotide transcripts in binding studies of receptor presence in target tissues. In very interesting and fundamental work in the field of cholinergic control of biological rhythms, Dojo et al. (2017) described carbachol-induced phase shifts of Per1 rhythms. The authors used cultured suprachiasmatic nucleus (SCN) slices to overcome problems with intracerebroventricular carbachol treatment. Previously, intracerebroventricular carbachol treatment was shown to cause phase delays during the subjective early night and phase advances in the subjective late night [see (Dojo et al., 2017) for details]. Dojo *et al.* found a carbachol-induced biphasic effect on the phase shift, which was blocked by atropine but not mecamylamine (nicotinic blocker). Thus, the authors concluded that these phase shifts are mAChR-dependent and used *in situ* hybridization to detect mAChR subtypes, finding M_3_ and M_4_ mAChRs expressed in SCN cells. The first effect that cannot be excluded concerning atropine properties is antagonism at nicotinic receptors (see Section 3.1). Although mecamylamine failed to block the phase shift, it is necessary to stress that mecamylamine blocks only some nicotinic receptors or subunits (α_3_ with p_IC50_ = 6.4 and α_4_ with IC_50_ = 5.3–6.5) (Alexander S. P. H. et al., 2017), while atropine blocks not only mAChRs but also the α_5_ subunits of nicotinic receptors and other receptors (see above—α_2A_- and α_1D_-adrenoceptors, glycine receptors, and SERTs 5-HT_1A_ and 5-HT_2C_). Thus, the exclusion of nicotinic receptors cannot be absolute. Additionally, other receptors could be affected by the 100 μmol l^−1^ (10^−4^ mol l^−1^) atropine concentration used in this experiments. α_4_-containing NRs (which form heteropentameric NRs) have a high affinity for nicotine and are the most frequent receptor subtype in both the rodent and human brains (Weinhart et al., 2021). The α_5_ subunit does not directly contribute to the agonist-binding site but plays a critical role in the functional properties of the receptor (Weinhart et al., 2021). Concerning mAChR subtypes, by *in situ* hybridization, the authors found that M_3_ and M_4_ mAChRs are expressed in SCN cells. However, no M_4_ mAChRs were found in the SCN (Valuskova et al., 2018b) in KO animals, and the overall density of mAChRs in the SCN was low. The general problem with the method of using nucleotide sequence detection is that this method localizes specific nucleotide transcripts within a portion or section of tissue but does not detect the presence of target proteins or binding sites.

As the last example, we would like to demonstrate the role of mAChRs in addiction. The study by (Wu et al., 2020) described the role of the rostromedial tegmental nucleus (also known as the tail of the ventral tegmental area) in mAChR-regulated opioid addiction. For the identification of mAChRs involved in opioid modulation of rostromedial tegmental GABAergic neurons, the authors used pilocarpine, 4-DAMP, LY2033298, and tropicamide. They concluded that M_3_ mAChRs are responsible for the opioid modulation of GABAergic neurons. In detail, pilocarpine (considered an M_3_ mAChR agonist) inhibited the acquisition of morphine-induced conditional place preference, and 4-DAMP (considered an M_3_ mAChR antagonist) reversed the inhibitory effect of pilocarpine. 4-DAMP also increased locomotor activity, while pilocarpine partially decreased locomotor activity (when combined with morphine). LY2033298 is usually considered an M_4_-selective agonist and has a more complicated mode of action—it also binds to M_2_ mAChRs and mediates both positive and negative allosteric effects, depending on the orthosteric ligand. More concretely, it is a PAM of oxotremorine-M signaling at the M_2_ mAChR but a NAM of xanomeline at the same receptor. LY2033298 and tropicamide (considered an M_4_ mAChR antagonist) did not affect the acquisition of morphine-induced conditional place preference or locomotor activity. As discussed above, pilocarpine, which is declared an M_3_ selective agonist, has comparable effects on all mAChR subtypes, and 4-DAMP, which is known as an M_3_ mAChR-selective antagonist, reveals similar affinity to M_4_ mAChRs. Tropicamide is a drug with a similar affinity to all mAChRs and is also far from selective in blocking M_4_ mAChRs. The exact numbers of M_3_ and M_4_ mAChRs in the rostromedial tegmental area are not known. Thus, we can use the numbers of these mAChRs in the midbrain as reference values, although with reservation. According to (Oki et al., 2005), 38% of receptors in the midbrain are M_3_ mAChRs, and no significant number of M_4_ mAChRs are present. However, this study (Oki et al., 2005) experienced an important problem: in some cases, the total number of mAChRs when summing the numbers of specific mAChR subtypes exceeded the number of mAChRs in WT animals. Thus, one should be cautious in relying on the respective percentages of specific mAChR subtypes. The most abundant subtype in the midbrain is M_2_ mAChRs. Thus, it should be concluded that the role of M_3_ mAChRs in mAChR-regulated opioid addiction is highly probable, but we cannot exclude the roles of other mAChR subtypes (M_2_ mAChRs).

## 6 Discussion

**FIGURE 1:**
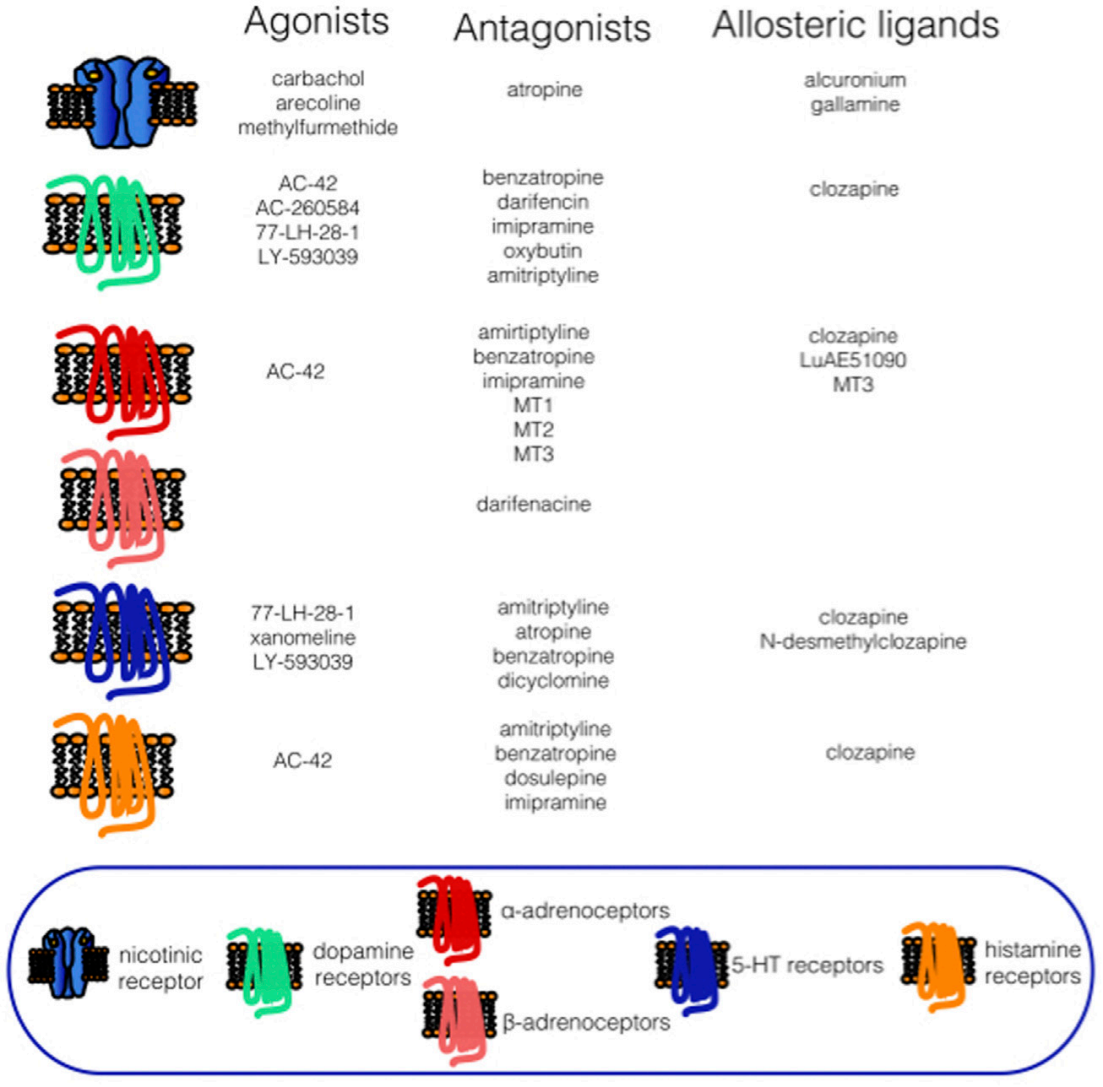
The main receptor targets of muscarinic agonists, antagonists and allosteric ligands. See the legend below.

We have shown in the previous sections that both agonists and antagonists and some allosteric ligands do not have single target (see Figure 1). Thus, the muscarinic drugs can be considered “multitargeting.”

Importantly, the pharmacological data are not identical, and one should also consider which constant is given. From that point of view, the correct assessment (proper steady-state) of the inhibition constant (K_i_) is important information. Half-maximal inhibitory concentration (IC_50_) is not a good predictor of pharmacodynamics properties. It does not account for many factors in drug action (Lamba and Pesaresi, 2022).

This type of drug action is not specific to mAChRs but is, at least in some cases, an advantage of drugs, allowing them to affect more pathway changes in specific diseases (Abatematteo et al., 2021). Surprisingly, we can find that ligands believed to specifically bind to one neurotransmitter receptor type had much wider activities. For example, dopamine receptor ligands have also a wide spectrum of targets (Myslivecek, 2022). This is just one example of such similar affinities to different neurotransmitter receptors, and many others can be found, which indicates that binding ligands that are believed to be specific to other neurotransmitter receptors are a general property of neurotransmitter receptors. Compared to other cholinergic targets, mAChRs are less selective in their orthosteric binding sites; thus, the selection of drugs for mAChR functions must involve more caution.

The use of mAChR agonists and/or antagonists in research on physiological/pathophysiological functions should thus include caution. When choosing a ligand for mAChR subtype determination, one should consider the limited selectivity. As it can be deduced from ligand structures, the ligands have, in many cases, similar structures. Together with the high degree of amino acid sequence homology, this is the reason for the difficulties in subtype function determination.

**FIGURE 2:**
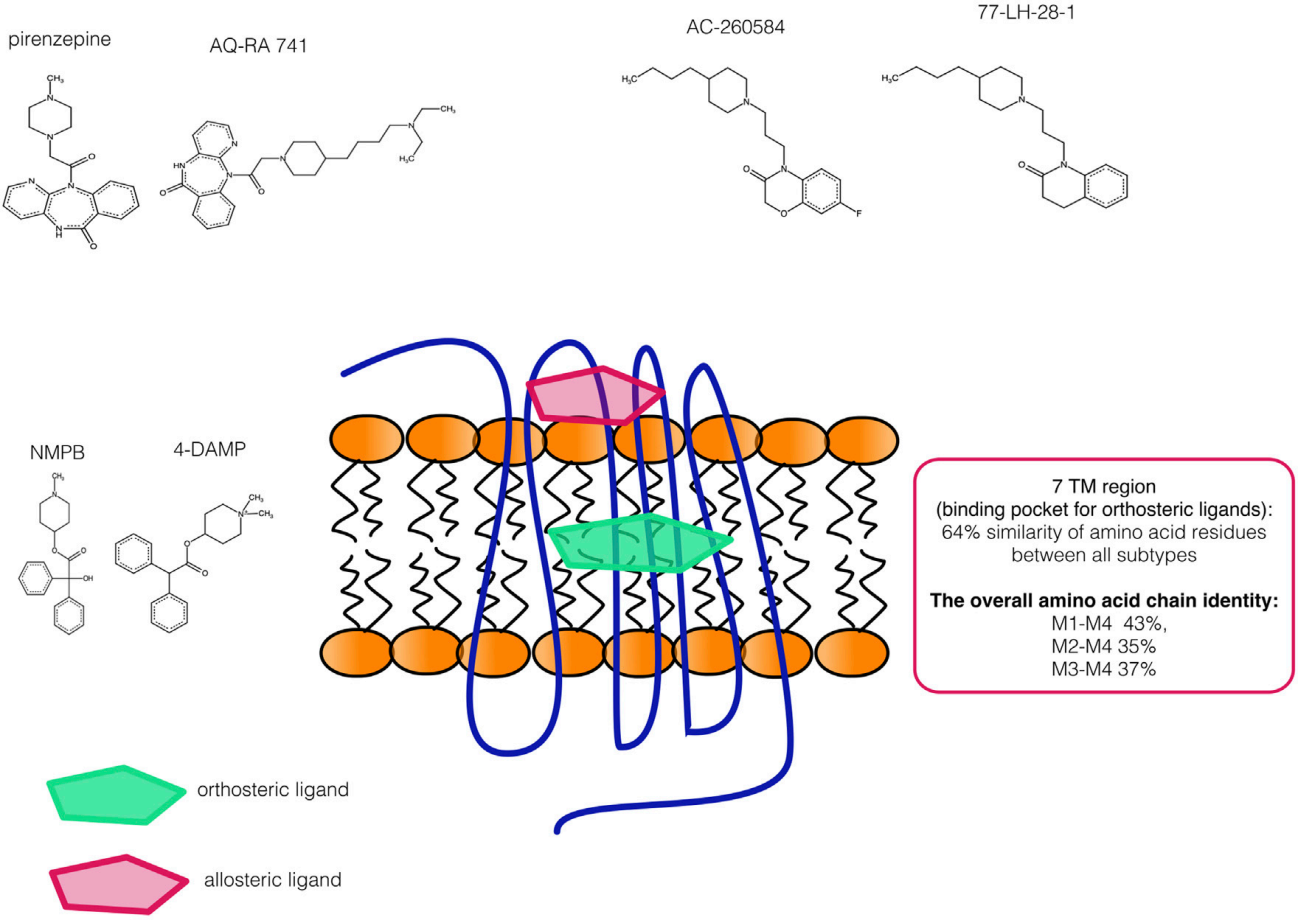
The schematic representation of selectivity problem with muscarinic orthosteric ligands. The binding of orthosteric ligands to transmembrane (TM) zones and binding site of allosteric ligands is also sechamatically shown. The similarity in amino acid chain is shown in the frame. Three examples of ligands with similar structure demonstrate that not only amino acid similarity but also ligand structural similarity are the basis of selectivity problems.

This structural similarity is one of the causes of subtype similarity in orthosteric ligands binding (see Figure 2). mAChR subtypes are mainly conserved within seven transmembrane zones - the structure to which ligands are bound (e.g., there is approximately 64% of identity between all mAChR subtypes, i.e., M_1_-M_5_ (Peralta et al., 1987; Bonner, 1989). In other words, 64% of amino acids are the same in all mAChRs. Thus, if the identical amino acid residue is present in the same position between mAChR subtypes, then the binding of ligand could be similar to mAChR subtypes. This can be demonstrated by similarities of binding for different substances revealed by crystal structure description (Thal et al., 2016). This high degree of sequence conservation does not exclude that orthosteric ligands can differently bind thanks to differences in tertiary structure. The molecular distinction is present in the third intracellular loop (participating in the G protein interactions) between odd- and even-numbered mAChRs (Caulfield and Birdsall, 1998). The overall amino acid chain identity display 43, 35, and 37% between M_4_ mAChR and M_1_, M_2_, and M_3_ mAChR, respectively (Peralta et al., 1987). In detail, the comparison of the M_1_, M_2_, M_3_, and M_4_ tiotropium-bound structures (using crystallization) with the structurally similar ligand QNB, revealed considerable differences around residues D3.32, Y7.39, and Y7.43 (Thal et al., 2016), interacting with an amine group. Similarly, other structure–binding properties are discussed in this review (Thal et al., 2016). Recently, the cryo-electron microscopy structures of M_1_ and M_2_ mAChR was described (Maeda et al., 2019), the crystal structure of M_5_ mAChR (Vuckovic et al., 2019), as well as structural insights into the subtype-selective antagonist binding to the M_2_ mAChR (Suno et al., 2018).

In detail, the similarities in orthosteric ligand structures as seen in Figure 2 are one reason for the limited selectivity of ligands. It is possible to demonstrate this by the effort to substitute the part of chains in mAChR ligand structure with other residues (methyl, benzyl, and others) that significantly change the properties of molecules. For examples of these efforts see data on the synthesis of selective ligands (e.g., (Melancon et al., 2012a; Salovich et al., 2012; Gentry et al., 2013a; Gentry et al., 2013b; Le et al., 2013b; Beshore et al., 2018; Nabulsi et al., 2019)). The second reason is the similarities in amino acid residues structure. For example (Suno et al., 2018), have constructed the thermostabilized mutant of M_2_ mAChRs (S110R) that had a significantly lower affinity for the agonist iperoxo than the wild type, whereas the affinity was unchanged or slightly lower for the inverse agonists (QNB, tiotropium and NMS). There was also difference in binding of antagonists (AF-DX 384 and pirenzepine), versus the inverse agonists with this mutation. This suggest that antagonists bind the M_2_ mAChR in an allosteric sodium ion–dependent manner, whereas inverse agonists do not. In contrast to these ligands, AFDX-384 had a remarkably higher affinity for the S110R mutant. It is notable that AFDX-384 is bound by M_2_ mAChR than pirenzepine (see Table 2). Another point is the conformations of critical residues that are needed for the receptor activation (Maeda et al., 2019). These motifs (see (Maeda et al., 2019) for details) are also similar in active conformations of M_1_ mAChR and M_2_ mAChR. This suggests that the activation mechanism is shared between M_1_ and M_2_ mAChRs, although these receptors are coupled with different G-proteins. (Maeda et al., 2019). Similarly, the amino acid residues, responsible for allosteric binding have been also identified (or more exactly are gradually identified). For example, key residues involved in the activity of BQCA, including Y179 in the second extracellular loop and W4007.35 in TM7, were critical for the activity of all PAMs tested by (Khajehali et al., 2018). This indicates that structurally distinct PAMs share a similar binding site with BQCA. More specifically, an extracellular allosteric site is defined by residues in TM2, TM7 and ECL2 (Khajehali et al., 2018). Similarly (Abdul-Ridha et al., 2014), have optimized the synthesis of benzoquinazolinone 12. They also fully characterized the pharmacology of this drug, finding that its improved potency derived from a 50-fold increase in allosteric site affinity as compared with BQCA, while retaining a similar level of positive cooperativity with acetylcholine.

Important data on the interaction of allosteric compounds with M_4_ mAChRs were reported by (Wang et al., 2022), where the binding of newly synthesized compound-110, iperoxo, or iperoxo-LY2119620 to M_4_ mAChR-G_i_ complex was studied. The authors described different interaction modes and activation mechanisms of M_4_ mAChR, and the receptor-iperoxo -LY-2119620-G_i_ cooperativity.

In this review, we tried to include as many ligands as possible for which the relevant off-target were found. The compounds with not clearly defined targets or those that do not target CNS-relevant molecules were excluded.

Most importantly, one should notice that ligands targeting similar (or same structures) reveal also structural similarity. For example, AC-260584 and 77-LH-28-1 (Heinrich et al., 2009) reveal structural similarities (see Figure 2) and can affect D_2_ DR in nanomolar concentration (see Table 1). In detail, K_i_ for D_2_ DR is 0.05, and 0.06, respectively, for AC-260584 and 77-LH-28-1 (Heinrich et al., 2009). Similarly, the structural similarity of atropine and bezatropine could be the reason for nanomolar affinity (pK_i_ = 6.28, and pK_i_ = 7.49) for atropine and benzatropine, respectively) of 5-HT_2C_ serotonin receptors to these ligands (Modell et al., 1989; Gaulton et al., 2016).

To overcome the limited selectivity of orthosteric ligands, it is recommended to carefully study the presence of respective subtypes in specific tissues via KO studies. Two studies conducted on mice with specific deletions of mAChR subtypes can serve as references. The first study by Ito et al., employed KO animals to determine the mAChR subtype numbers in peripheral tissues, such as the salivary glands, lung, heart, stomach, pancreas, bladder, and prostate (Ito et al., 2009). The second study by Oki et al., employed KO animals to investigate the presence of mAChR subtypes in specific areas in the CNS (Oki et al., 2005), including the cerebral cortex, corpus striatum, hippocampus, hypothalamus, thalamus, midbrain, pons-medulla, cerebellum, and spinal cord. Although one may assume that this type of study would provide a clear picture of the presence of mAChR subtypes in specific tissues, a compensatory mechanism also exists—if one subtype is knocked down, the presence of the remaining subtypes in the specific tissue would increase. In fact, an additional problem can also lie in the methodology. (Oki et al., 2005) and (Ito et al., 2009) used direct radioligand binding with hydrophilic ligands in tissue homogenates, which can slightly artificially increase binding. This problem can be overcome by using autoradiography, in which binding can be determined in very small brain areas (Farar and Myslivecek, 2016). Another method reported by (Lebois et al., 2018) employed the detection of mRNA transcript levels expressed as reads per kilobase per million mapped reads. These results are in better agreement with our data obtained using autoradiography.

Thus, the total amount of all mAChR subtypes (i.e., the sum of the density of M_1_+M_2_+M_3_+M_4_+M_5_ mAChRs), as reported by (Oki et al., 2005; Ito et al., 2009), could exceed the density of all receptor subtypes determined in WT animals. For example, in the submandibular gland (Ito et al., 2009), 35% M_2_ mAChRs and 79% M_3_ mAChRs were found, totaling 114%. It is, therefore, necessary to consider that the major subtype is M_3_ mAChRs, which are present at more than 70%, and that the second subtype, M_2_ mAChRs, is present at slightly less than half of the number of M_3_ mAChRs (i.e., the ratio is approximately 2:1). To present another example of how knocking down a specific subtype can increase the numbers of other subtypes, according to (Oki et al., 2005), 45% of M_1_ mAChRs, 34% of M_2_ mAChRs, 19% of M_3_ mAChRs, and 54% M_4_ mAChRs are present in the striatum. These numbers total 152% (the total should be 100%). Thus, it is better to count the subtypes according to the M_1_:M_2_:M_3_:M_4_ ratio, which is 20:22:13:35. According to our measurements using autoradiography (Valuskova et al., 2018a), 37% M_1_ mAChRs and 46% M_4_ mAChRs were found, while we did not detect M_2_ mAChRs. Unfortunately, we did not have M_3_ (or M_5_)-KO animals at our disposal. Although the numbers are different, it is evident that the most abundant subtype in the striatum is M_4_ mAChRs and that a substantial number of M_1_ mAChRs are present. Recently, we proposed an algorithm for subtype determination (Myslivecek, 2021). Here, we would like to provide the reader with a more detailed procedure for subtype identification. First, as described above, it is necessary to establish a rough ratio of the mAChR subtypes present in the tissue of interest. Let us use the example of the striatum, with an M_1_:M_4_ ratio between 36-45:55-64. The second step is the selection of a ligand able to discriminate between M_1_ mAChRs and M_4_ mAChRs. This criterion is partly fulfilled by antagonist PD102807 (the orthosteric site does not provide enough selectivity—see Section 3), which has a selectivity for M_4_ mAChRs over 2 grades of magnitude higher than that for M_1_ mAChRs (pKi = 7.3–7.4 vs. pKi = 5.3–5.5; see (Myslivecek, 2019)). As mentioned above, to date, no interactions with other targets are known. Thus, the third step, which is the most difficult, is the choice of the antagonist concentration. The pKi defines the concentration at which a ligand binds to 50% of available binding sites. In our case, the pKi = 7.3–7.4 indicates approximately 39.8–50.1 nmol/L. This means that PD 102807 at a concentration of approximately 45 nmol/L will occupy approximately 50% of M_4_ mAChRs. Virtually no M_1_ mAChRs will be inhibited at this concentration (Bohme et al., 2002). The affinity of M_1_ mAChRs for PD 102807 is 72-fold lower than that of M_4_ mAChRs (Augelli-Szafran et al., 1998). This means that at concentrations approaching 1 μmol/L, all M_4_ mAChRs will be blocked, but almost no M_1_ mAChRs will be affected by this drug. However, the affinity of M_3_ mAChRs for this drug is higher (there is a 10-fold difference—see (Augelli-Szafran et al., 1998)) than that of M_2_ mAChRs by 38 fold. According to the possible ratios (Oki et al., 2005; Valuskova et al., 2018a), and assuming that the researcher uses PD 102807 at 1 μmol/L, all M_4_ mAChRs will be blocked, and almost no M_1_ or M_2_ mAChRs will be affected by this drug. M_3_ mAChRs are present at only one-third of the number of M_4_ mAChRs and have a 10-fold lower affinity for PD 102807. However, discriminating between M_3_ and M_4_ mAChRs is impossible, as no drug with an appropriate difference in selectivity is known. Thus, it is necessary to conclude that there is a possibility of minor involvement of M_3_ mAChRs (with respect to their 10-fold lower affinity for PD 102807 and one-third lower density in comparison to M_4_ mAChRs).

As additional examples (Oki et al., 2005), identified three mAChR subtypes in the hippocampus (M_1_-M_3_) and pons medulla (M_2_-M_4_), two mAChR subtypes in the hypothalamus and midbrain (M_2_-M_3_), and only M_2_ mAChRs in the cerebellum and spinal cord. If M_1_-M_3_ mAChR subtypes are present in the hippocampus, then it is possible to use pirenzepine, which has approximately one grade of magnitude higher selectivity for M_1_ mAChRs than M_2_ and M_3_ mAChRs. According to (Oki et al., 2005), 69% M_1_, 30% M_2_, and 17% M_3_ mAChRs were present, totaling 116%. The corrected ratio should thus be 59:26:15. On the other hand, we used tritiated pirenzepine for M_1_ mAChR determination and showed that in M_1_ mAChR-KO animals (Valuskova et al., 2018a), the binding of ^3^H-pirenzepine was almost completely abolished in hippocampal areas (dorsal hippocampus, CA1, CA3 area, and dentate gyrus). Examples of antagonists that could be used in specific areas according to the presence of mAChR subtypes are as follows: in the pons-medulla (M_2_-M_4_), PD12807 would identify M_4_ mAChRs, and in the hypothalamus and midbrain (M_2_-M_3_), methoctramine would identify M_2_ mAChRs.

As mentioned above, a new method for identifying mAChR subtypes involved in specific functions involves the use of allosteric ligands (PAMs and NAMs), as recently demonstrated in M_4_ and M_5_ mAChR determination as important structures in alcohol-seeking behaviors (Walker et al., 2021). Similarly, some new positron emission tomography (PET) tracers have exhibited promising selectivity (Ozenil et al., 2021).

## 7 Conclusion

The main conclusion of this review is that the cholinergic system in different peripheral/CNS functions should first be studied by searching for the mAChR subtype affinities of ligands of choice. Although it is sometimes difficult to differentiate between specific subtypes, there are some exceptions in which a ligand can be used as a specific means of mAChR subtype identification (Valuskova et al., 2018a). Another search should determine the respective densities of mAChR subtypes in specific tissues. The combination of these two searches should increase accurate conclusions concerning mAChR subtypes involved in specific functions.

One way to identify the subtypes involved is to use conditional KO animals. However, it is also necessary to keep in mind that targeting one receptor can affect the functions of other receptors. Typically, this is the case for mAChRs vs. adrenoceptors in the heart (Myslivecek et al., 1996; Myslivecek et al., 1998; Myslivecek et al., 2004; Myslivecek et al., 2008c; Benes et al., 2012; Laukova et al., 2014; Tomankova et al., 2015). Moreover, conditional, tissue-specific KO is a better option than classical KO. The finding that muscarinic agonists or antagonists have a multitarget nature is not unique, and one should be aware that this can be true for many GPCR agonists/antagonists.

Importantly, limited binding selectivity for specific neurotransmitter receptors is not a property relevant to only mAChRs but is also a general attribute of most neurotransmitter receptors (see the examples for dopamine receptors in the Discussion).
